# Bifactor exploratory structural equation modeling: A meta-analytic review of model fit

**DOI:** 10.3389/fpsyg.2022.1037111

**Published:** 2022-10-26

**Authors:** Andreas Gegenfurtner

**Affiliations:** Methods in Learning Research, University of Augsburg, Augsburg, Germany

**Keywords:** factor analysis, meta-analysis, multidimensionality, goodness-of-fit, exploratory structural equation modeling, bifactor ESEM

## Abstract

Multivariate behavioral research often focuses on latent constructs—such as motivation, self-concept, or wellbeing—that cannot be directly observed. Typically, these latent constructs are measured with items in standardized instruments. To test the factorial structure and multidimensionality of latent constructs in educational and psychological research, Morin et al. (2016a) proposed bifactor exploratory structural equation modeling (B-ESEM). This meta-analytic review (158 studies, *k* = 308, *N* = 778,624) aimed to estimate the extent to which B-ESEM model fit differs from other model representations, including confirmatory factor analysis (CFA), exploratory structural equation modeling (ESEM), hierarchical CFA, hierarchical ESEM, and bifactor-CFA. The study domains included learning and instruction, motivation and emotion, self and identity, depression and wellbeing, and interpersonal relations. The meta-analyzed fit indices were the χ^2^*/df* ratio, the comparative fit index (CFI), the Tucker-Lewis index (TLI), the root mean square error of approximation (RMSEA), and the standardized root mean squared residual (SRMR). The findings of this meta-analytic review indicate that the B-ESEM model fit is superior to the fit of reference models. Furthermore, the results suggest that model fit is sensitive to sample size, item number, and the number of specific and general factors in a model.

## Introduction

To examine the factorial structure and multidimensionality of latent constructs in educational and psychological research, Morin et al. (2016a) proposed bifactor exploratory structural equation modeling (B-ESEM) as a methodological synergy that integrates bifactor modeling and exploratory structural equation modeling. Since their seminal paper, a number of studies have applied B-ESEM in research on learning and instruction, motivation and emotion, self and identity, wellbeing, and other areas. The present systematic review and meta-analysis aimed to collect and describe these studies, meta-analyze their reported model fit, and estimate the extent to which the fit of the B-ESEM model differs from that of the other tested model representations. A secondary aim was to analyze how sensitive model fit was to sample size, item number, and the number of specific and general factors in a model.

### Exploring the factor structure of multidimensional constructs

**Figure 1:**
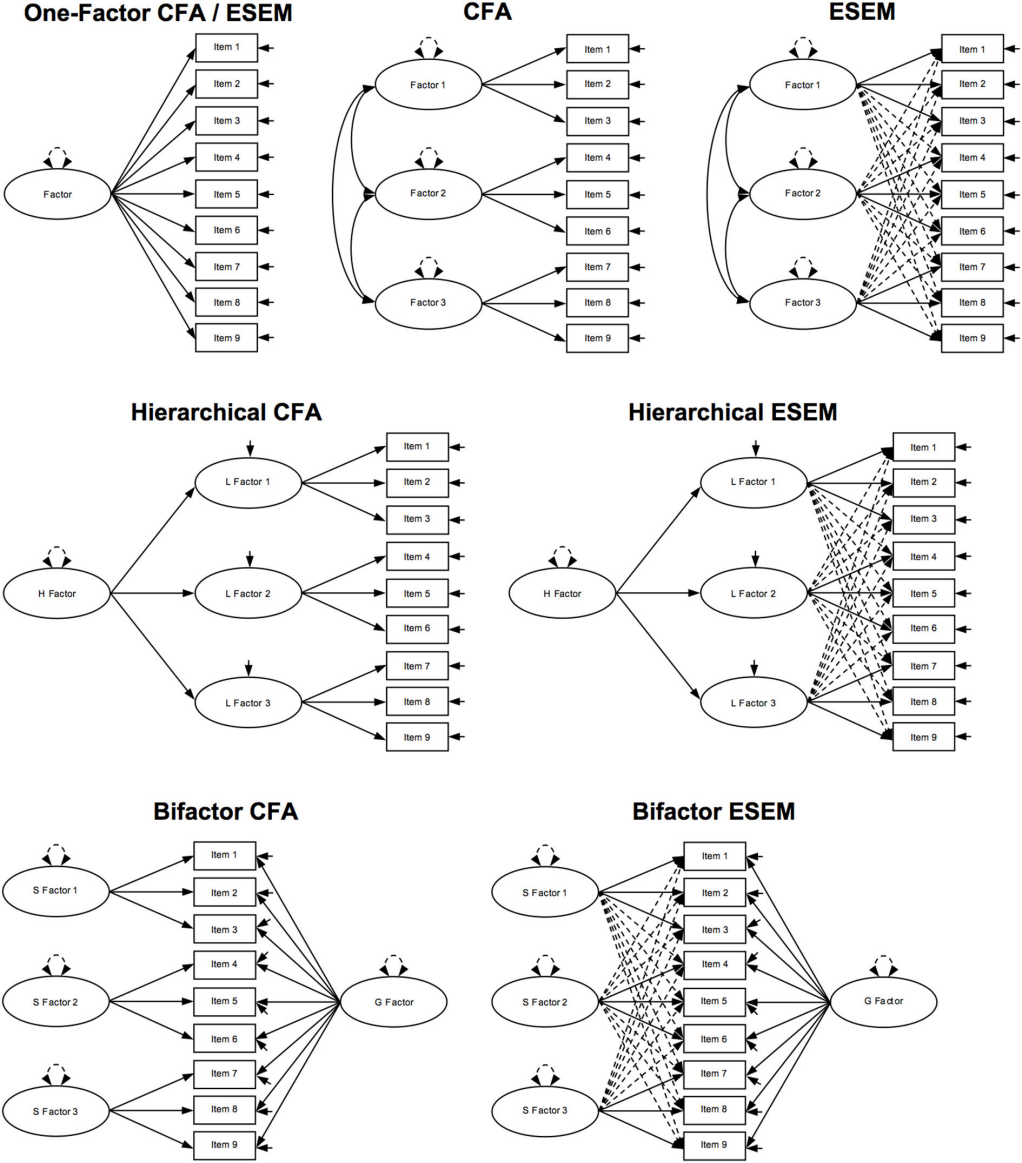
Schematic model representations. Ovals represent latent factors; squares represent observed variables; full unidirectional arrows between ovals and squares represent factor loadings; dotted unidirectional arrows between ovals and squares represent cross-loadings; full unidirectional arrows linked to a single oval represent factor disturbance; full unidirectional arrows linked to a single square represent item uniqueness; bidirectional full arrows between ovals represent factor covariances/correlations; bidirectional dashed arrows linked to a single oval represent factor variance; CFA, confirmatory factor analysis; ESEM, exploratory structural equation modeling; H Factor, higher-order factor in a hierarchical model; L Factor 1–3, lower-order factors in a hierarchical model; S Factor 1–3, specific factors in a bifactor model; G Factor, general factor in a bifactor model.

Researchers interested in exploring the factorial structure of a multidimensional construct can choose among analytical options including confirmatory factor analysis (CFA; Jöreskog, 1969), exploratory structural equation modeling (ESEM; Asparouhov and Muthén, 2009), and hierarchical and bifactor representations of CFA and ESEM (Rindskopf and Rose, 1988; Reise, 2012; Morin et al., 2013, 2016a). A number of excellent review papers and pedagogical illustrations of these factor analytic techniques exist (Marsh, 2007; Marsh et al., 2009, 2014; Reise, 2012; Morin et al., 2013, 2016a, 2020; Bandalos and Finney, 2018; Sellbom and Tellegen, 2019). Interested readers can consult these excellent resources for more detail, but let us briefly review how the factor structure of multidimensional latent constructs can be represented and analyzed. Figure 1 offers an overview of schematic model representations.

The simplest form of representation and analysis, arguably, is a one-factor first-order model in which all items are loaded on a single factor. More complex are CFA and ESEM that can be applied to test a hypothesized factor structure: in CFA, items load on a single factor with zero cross-loadings; in ESEM, too, items load a single factor but without CFA's strict requirement of zero cross-loadings. When exploring hierarchically ordered constructs, CFA and ESEM can be extended to models in which items are loaded on specific lower-order factors (L-factors), which are in turn loaded on a single higher-order factor (the H-factor). Finally, in the case of bifactor modeling, CFA and ESEM can be extended to a model in which items are loaded on one general factor (the G-factor) representing a global overarching construct as well as on multiple specific factors (S-factors) representing their subscales—in bifactor CFA (B-CFA) without and in bifactor ESEM (B-ESEM) with free estimation of cross-loadings between items and non-target factors. B-ESEM in particular has been shown to result in a better model fit for a number of multidimensional hierarchical constructs that are frequently explored in educational and psychological research^1^.

### B-ESEM in multivariate behavioral research

A growing number of studies test the multidimensionality of latent constructs in research on learning and instruction, motivation and emotion, self and identity, wellbeing and depression, and interpersonal relationships. In the domain of learning and instruction, for example, Scherer et al. (2016) identified the presence of a general factor of student-perceived instructional quality and three distinct sub-dimensions: teacher support, cognitive activation, and classroom management. Fernandez-Rio and colleagues explored the factor structure of a scale measuring dimensions of cooperative learning. In the domain of motivation and emotion, Howard et al. (2020) showed that motivation scales based on the self-determination theory were best represented as a B-ESEM model in which all items defined specific motivation regulatory qualities on a continuum (from amotivation to intrinsic motivation) and were also used to define a global self-determination factor. Perera et al. (2018b) tested the structure of teacher engagement, identifying a general factor and specific cognitive-physical, emotional, and social engagement factors. In the domain of self and identity, for example, Arens et al. (2021) explored the structure of academic self-concept and reported that a B-ESEM representation including one general self-concept factor and multiple specific self-concept factors (for German, English, math, physics, chemistry, biology, and history) provided the best model fit compared with reference models. In the domain of wellbeing and depression, Morin et al. (2017) tested the dimensionality of the Index of Psychological WellBeing at Work that can also be used to wellbeing of teachers. In the domain of interpersonal relations, Ratelle et al. (2018) showed that the factor structure of a scale measuring parental structure, including parents' rules, predictability, feedback, opportunities, rationale, and authority, was best represented as a B-ESEM model.

These individual studies support the assumption that B-ESEM is best used to deal with multidimensional hierarchical constructs, yet the extent to which model fit of B-ESEM differs from other model representations has not yet been investigated across studies, domains, and scales. A meta-analysis of published studies could help to close this research gap. Among the frequently used parameters used in the studies reviewed to test model fit are the χ^2^*/df* ratio (Pearson, 1900), the comparative fit index (CFI; Bentler, 1990), the Tucker-Lewis index (TLI; Bentler and Bonett, 1990), the root mean square error of approximation (RMSEA; Steiger, 1990), and the standardized root mean squared residual (SRMR; Bentler, 1995). Table 1 provides an overview of goodness-of-fit indices used in B-ESEM studies^2^. Furthermore, if the evidence that model fit indices are sensitive to sample size, item number, and factor number from simulation studies is valid (Hu and Bentler, 1999; Marsh et al., 2004; Shi et al., 2019), it would be interesting to replicate these findings through a research synthesis using real data rather than simulated data. Such a synthesis would also be useful to describe the status quo of research adopting a B-ESEM framework, the mapping of tested constructs (such as academic self-concept; Arens et al., 2021), scales (such as the Multidimensional Work Motivation Scale; Howard et al., 2020), and domains (such as learning and instruction).

**Table 1:** Factor analytic techniques and model fit indices.

	**Abbreviation**	**References**
**Factor analytic techniques**		
Confirmatory factor analysis	CFA	Jöreskog, 1969
Exploratory structural equation modeling	ESEM	Asparouhov and Muthén, 2009
Hierarchical confirmatory factor analysis	H-CFA	Rindskopf and Rose, 1988
Hierarchical exploratory structural equation modeling	H-ESEM	Morin et al., 2013
Bifactor confirmatory factor analysis	B-CFA	Reise, 2012
Bifactor exploratory structural equation modeling	B-ESEM	Morin et al., 2016a
**Model fit indices**		
Chi square/degrees of freedom ratio	χ^2^/*df*	Pearson, 1900
Comparative fit index	CFI	Bentler, 1990
Tucker-Lewis index	TLI	Bentler and Bonett, 1990
Root mean square error of approximation	RMSEA	Steiger, 1990
Standardized root mean squared residual	SRMR	Bentler, 1995

### The present study

Based on a systematic literature review and meta-analysis of previous research applying the B-ESEM framework, the present study compares goodness-of-fit indices of B-ESEM with reference models. The study sought to answer three research questions: (1) Which domains, constructs, and scales are targeted in studies adopting a B-ESEM framework? (2) What is the model fit of B-ESEM representations compared to CFA, ESEM, H-CFA, H-ESEM, and B-CFA representations? (3) How sensitive is model fit to sample size, item number, and the number of specific and general factors in a model?

## Methods

Steps in conducting and reporting this meta-analytic review of B-ESEM included defining inclusion and exclusion criteria, searching the literature, coding information from the retrieved studies, calculating intercoder reliability, and meta-analyzing the fit indices (Page et al., 2021). The following sections describe these steps in more detail.

### Defining inclusion and exclusion criteria

We identified studies that reported goodness-of-fit indices of B-ESEM in educational and psychological research. Table 2 presents the criteria for inclusion and exclusion of these studies. To be included, a study had to report a χ^2^ (df), CFI, TLI, RMSEA, or SRMR estimate of a bifactor ESEM model and compare it with a CFA, ESEM, H-CFA, H-ESEM, or B-CFA reference model. Studies were omitted if they did not examine a B-ESEM representation, did not compare B-ESEM to a reference model, or relied on simulated data. To minimize publication bias, our literature search was deliberately broad and included reports in journal articles, book chapters, monographs, conference papers, and unpublished theses or dissertations, regardless of participant population, publication language, publication year, or domain.

**Table 2:** Criteria for inclusion and exclusion in the meta-analytic review.

**Criterion**	**Inclusion**	**Exclusion**
Model representation	B-ESEM	No B-ESEM
Reference model	CFA, ESEM, H-CFA, H-ESEM, or B-CFA	No reference model
Model fit index	χ^2^ (*df*), CFI, TLI, RMSEA, or SRMR	No χ^2^ (*df*), CFI, TLI, RMSEA, or SRMR
Data type	Original data	Simulated data
Study type	Original research	Method reviews, meta-analyses, errata, editorials
Publication type	All publication types, including journal articles, theses, monographs, chapters, conference proceedings	–
Publication language	All languages	–
Publication year	All years	–
Population	All populations	–
Domain	All domains	–

B-ESEM, bifactor exploratory structural equation modeling; CFA, confirmatory factor analysis; ESEM, exploratory structural equation modeling; H-CFA, hierarchical confirmatory factor analysis; H-ESEM, hierarchical exploratory structural equation modeling; B-CFA, bifactor confirmatory factor analysis; CFI, comparative fit index; TLI, Tucker-Lewis index, RMSEA, root mean square error of approximation, SRMR, standardized root mean squared residual.

To illustrate how we applied the criteria, examples of both excluded and included articles may help. On the one hand, an excellent study by Zhu et al. (2020) that reports model fit evaluation of a B-ESEM representation of mindfulness was excluded because B-ESEM was not compared with other models; rather, the B-ESEM factor scores were used to perform latent profile analyses. On the other hand, a study by Arens et al. (2021) was included because the authors compared goodness-of-fit indices obtained from higher-order and bifactor ESEM representations of the structure of academic self-concept.

### Searching the literature

Grounded in the inclusion criteria presented in Table 2, a two-phase literature search was performed. First, we conducted an electronic search of five databases—ERIC, PsycINFO, PubMed, Scopus, and Web of Science—for all publication types in any language published up to December 31, 2021 and with relevant keywords in the title or abstract. The keywords were *bifactor exploratory structural equation modeling, bifactor ESEM*, and *B-ESEM*, along with their spelling variants. This database search resulted in a total of 525 hits: 25 from ERIC, 106 from PsycINFO, 34 from PubMed, 182 from Scopus, and 178 from Web of Science. Two trained raters eliminated 318 duplicates because they were listed in more than one database, after which 207 articles remained. Next, both raters independently and in duplicate screened a random subset of the 207 identified articles (10 percent; *n* = 21 articles). As interrater agreement was high, with Cohen's κ = 0.881 (95% CI = 0.659–1.00), a single rater continued to screen the remaining studies for eligibility by reading titles and abstracts, after which 186 articles remained. Both raters then read the full texts of these articles independently to check for eligibility, and 60 studies were excluded because they did not examine a B-ESEM representation (37 removals) or did not compare B-ESEM to a reference model (23 removals), resulting in 126 articles for inclusion in the meta-analytic review.

Second, we performed a forward cross-referencing search, using Google Scholar to identify studies that cited the influential article of Morin et al. (2016a) up until December 31, 2021. We read the titles and abstracts of the 723 citing studies and retrieved the full texts of 169 of these reports. After the full texts were read, 137 records were excluded because the studies did not examine a B-ESEM representation (129 removals), did not include a reference model (4 removals), or used simulated data (4 removals). The remaining 32 studies met all inclusion criteria and were thus included in the review.

**Figure 2:**
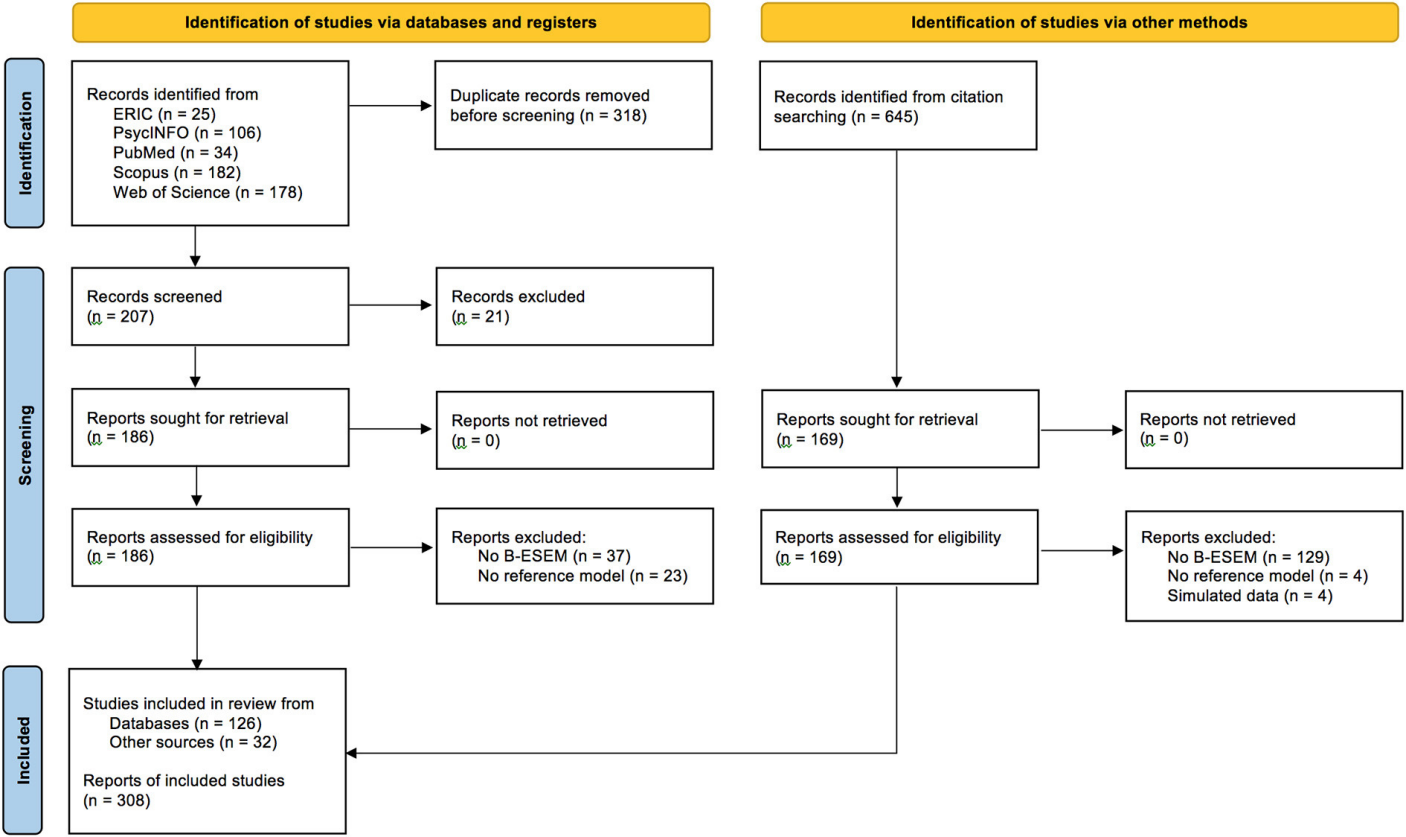
PRISMA study selection flow chart.

In summary, the literature search resulted in a total of 158 studies: 126 from the database search and 32 from the forward search. Figure 2 presents the PRISMA study selection flow diagram (Page et al., 2021). The included studies reported 308 comparisons of B-ESEM representations to reference models, which were subsequently coded.

### Coding information from the retrieved studies

Once the studies for inclusion were selected, two trained raters used the coding scheme presented in Table 3 to code a random subset of the 308 identified reports (10 percent; *n* = 31 reports) independently and in duplicate. Coded information included publication characteristics, measurement characteristics, and goodness-of-fit indices. Publication characteristics were coded as the first author and publication year. Measurement characteristics were coded as sample size, target construct, and scale name and abbreviation, as well as the number of items, specific factors, and general factors. Goodness-of-fit indices were coded separately for one-factor, CFA, ESEM, H-CFA, H-ESEM, B-CFA, and B-ESEM model representations; indices included CFI, TLI, RMSEA, SRMR, and the χ^2^*/df* ratio.

**Table 3:** Coding scheme.

**Main category**	**Sub-Category**	**Anchor example**
**Publication characteristics**		
Author	Name of first author	Haugen
Publication year	Coded as year	in press
**Measurement characteristics**		
Sample size	Sample size *N*	333
Construct	Target construct	Group cohesion
Scale properties	Scale name and abbreviation	Group environment questionnaire (GEQ)
	Number of items used	18
	Number of specific factors	4
	Number of general factors	1
**Goodness-of-Fit indices**		
One-factor	*χ^2^*	488.153
	*df*	135
	CFI	0.831
	TLI	0.810
	RMSEA	0.089
	SRMR	0.063
CFA	*χ^2^*	291.702
	*df*	129
	CFI	0.922
	TLI	0.908
	RMSEA	0.062
	SRMR	0.049
ESEM	*χ^2^*	155.324
	*df*	87
	CFI	0.967
	TLI	0.943
	RMSEA	0.049
	SRMR	0.028
H-CFA	*χ^2^*	302.934
	*df*	130
	CFI	0.917
	TLI	0.903
	RMSEA	0.063
	SRMR	0.050
H-ESEM	*χ^2^*	153.571
	*df*	88
	CFI	0.969
	TLI	0.945
	RMSEA	0.047
	SRMR	0.028
B-CFA	*χ^2^*	295.590
	*df*	118
	CFI	0.915
	TLI	0.890
	RMSEA	0.067
	SRMR	0.053
B-ESEM	*χ^2^*	173.867
	*df*	73
	CFI	0.952
	TLI	0.899
	RMSEA	0.064
	SRMR	0.024

CFI, comparative fit index; TLI, Tucker-Lewis index, RMSEA, root mean square error of approximation, SRMR, standardized root mean squared residual. CFA, confirmatory factor analysis; ESEM, exploratory structural equation modeling; H-CFA, hierarchical confirmatory factor analysis; H-ESEM, hierarchical exploratory structural equation modeling; B-CFA, bifactor confirmatory factor analysis; B-ESEM, bifactor structural equation modeling.

To illustrate our coding decisions, examples how we applied the coding scheme may help. For instance, Rodrigues et al. (2020) reported the fit of model representations with (a) one general and two specific factors, (b) one general and six specific factors, and (c) two general and six specific factors for the Behavioral Regulation in Sport Questionnaire and the Behavioral Regulation in Exercise Questionnaire. Because this meta-analytic review aimed to estimate the influence of factor number on model fit, we coded each model separately. Similarly, Tóth-Király et al. (2019) reported the model fit of representations with one or two general factors of the Basic Psychological Need Satisfaction and Frustration Scale. We coded each representation separately to allow for comparison. Generally, model fit was coded separately for studies that tested models with varying numbers of specific and/or general factors (e.g., Arias et al., 2016; Rodenacker et al., 2017; Gu et al., 2020; Frutos, 2021; Yi et al., 2021) and with varying item numbers. For example, Bianchi and Verkuilen (2021) examined the Green et al. Paranoid Thoughts Scale (GPTS) in its original 32-item version, a revised 18-item version, and an 8-item version. Model fit for each version was coded independently to allow for estimation of the influence of item number on the goodness-of-fit indices. Model fit was also coded separately for studies that used more than one sample (e.g., Neff et al., 2019; Howard et al., 2020; Longo et al., 2020; Vaughan et al., 2020) or measurement time (e.g., Stenling et al., 2018; Cece et al., 2019; Garn et al., 2019; Neff et al., 2021b). Section Research question 1: Description of B-ESEM studies provides a complete description of all coded study information.

### Calculating interrater reliability

To calculate interrater reliability and agreement of the literature search and the literature coding, we used an intraclass correlation coefficient (ICC) for continuous scales and Cohen's kappa coefficient (κ) for nominal scales. Table 4 presents all estimates. ICC and κ estimates tend to be robust when there are exactly two raters, as in our case, who searched and coded the studies independently and in duplicate. Consensus was reached *via* discussion when coding conflicts emerged. Following the recommendations of Koo and Li (2016), ICC estimates were calculated together with their 95% confidence intervals based on a mean-rating (*k* = 2), absolute-agreement, two-way mixed-effects model which can generally be judged as moderate (0.50–0.75), good (0.75–0.90), and excellent (>0.90) reliability. Following recommendations of Landis and Koch (1977), standard errors of Cohen's κ were calculated to compute the 95% confidence intervals around κ which can generally be judged as moderate (0.41–0.60), substantial (0.61–0.80), and almost perfect (>0.80) agreement.

**Table 4:** Interrater reliability and agreement of the literature search and coding.

**Step**	**Category**	**Coefficient**	**Estimate**	**95% confidence interval**
Literature search	Identification	κ	0.990	0.981–0.999
	Screening	κ	0.881	0.659–1.000
	Eligibility	κ	1.000	1.000–1.000
Literature coding	Continuous	ICC	0.923	0.901–0.932
	Categorical	κ	0.976	0.727–1.000

κ, Cohen's kappa coefficient; ICC, intraclass correlation coefficient.

Analyses for the literature search indicated substantial to almost perfect agreement for identification, κ = 0.990 (95% CI = 0.981; 0.999), screening, κ = 0.881 (95% CI = 0.659; 1.000), and eligibility, κ = 1.000 (95% CI = 1.000; 1.000). Analyses for the literature coding were performed separately for the continuous and nominal variables: the continuous scales were publication year, number of items, number of specific factors, number of general factors, and all goodness-of-fit indices, with an ICC = 0.923 (95% CI = 0.901; 0.932); the remaining scales were nominal, with κ = 0.976 (95% CI = 0.727; 1.000). In summary, these estimates indicate almost perfect agreement (Landis and Koch, 1977) and excellent reliability (Koo and Li, 2016).

### Meta-analyzing the fit indices

We meta-analyzed the fit indices in two steps. First, we computed average estimates of the goodness-of-fit indices of each model. Mean differences between models were calculated using one-way analyses of variance. Missing values were deleted casewise. Second, we estimated the effects of sample size, item number, number of specific factors, and number of general factors on the fit indices using within-study and between-study analyses. Within-study analyses included (a) descriptive analyses of two-tailed Pearson correlation coefficients and (b) aggregated changes of model fit as a result of changes in B-ESEM model structure. Between-study analysis included (a) an outlier detection analysis using the median absolute deviation approach with the formulae reported in Miller's (1991) and Leys et al. (2013) very conservative threshold 3 and (b) an unrestricted weighted least squares meta-regression analysis (Stanley and Doucouliagos, 2017) to estimate the relative influence of sample size, item number, and factor number on model fit.

## Results and discussion

### Research question 1: Description of B-ESEM studies

Research question 1 asked, “Which domains, constructs, and scales are targeted in studies adopting a B-ESEM framework?” The 158 studies included in the present analysis offered a total of 308 reports of B-ESEM model fit. Total sample size was 778,624 participants, with a mean sample size of 2,528.00 (*SD* = 25,492.06). On average, B-ESEM models were composed of 24.62 items (*SD* = 17.23), 4.62 specific factors (2.96), and 1.05 general factors (0.26). B-ESEM representations were compared to representations from B-CFA in 221 reports, H-ESEM in 25 reports, H-CFA in 57 reports, ESEM in 266 reports, CFA in 265 reports, and a one-factor model in 123 reports. Concerning publication language, 151 of the 158 included studies were written in English, 4 in Spanish, and 1 each in French, German, and Hungarian. Concerning publication type, 154 of the 158 included studies were journal articles, 3 were doctoral theses, and 1 was an unpublished manuscript. Table 5 presents a description of all included studies clustered into six domains.

**Table 5:** Study description.

**Domain**	**Construct**	**Scale**	**Included studies**
**Learning and instruction**			
	Cooperative learning	Cooperative learning scale	Fernandez-Rio et al., 2022
	Instructional quality	Instructional quality scale (as measured in PISA)	Scherer et al., 2016
**Motivation and emotion**			
	Academic motivation	Academic motivation scale (AMS)	Dierendonck et al., 2022
			Guay and Bureau, 2018
			Guay et al., 2021
			Howard et al., 2020
			Kartal, 2020
			Litalien et al., 2017
		Academic self-regulation questionnaire (SRQ-A)	Gordeeva et al., 2020
	Sport motivation	Behavioral regulation in sport questionnaire (BRSQ)	Rodrigues et al., 2020
			Stenling et al., 2018
		Youth behavioral regulation in sport questionnaire	Cece et al., 2019
		Behavioral regulation in exercise questionnaire (BREQ)	Rodrigues et al., 2020
		Cuestionario tridimensional de competencia percibida	Méndez-Giménez et al., 2020
		Empowering and disempowering motivational climate questionnaire (EDMCQ)	Appleton et al., 2016
			Milton et al., 2018
	Work motivation	Multidimensional work motivation scale (MWMS)	Howard et al., 2018
			Howard et al., 2020
			Howard et al., 2021
		Transfer motivation questionnaire (TMQ)	Gegenfurtner and Quesada-Pallarès, 2022
		Faculty motivation to teach in higher education	Calkins, 2018
		Work-Related motivational orientations measure	Burk and Wiese, 2018
	Job satisfaction	Leader satisfaction assessment (LSA)	Sutherland, 2020
		Minnesota satisfaction questionnaire (MSQ)	Sutherland, 2020
	Affective commitment	Workplace affective commitment multidimensional questionnaire (WACMQ-S)	Perreira et al., 2018
	Work engagement	Utrecht work engagement scale (UWES-9)	Gillet et al., 2019
			Huyghebaert-Zouaghi et al., 2021a
			Huyghebaert-Zouaghi et al., 2022
	Teacher engagement	Engaged teacher scale (ETS)	Perera et al., 2018b
	Student engagement	Student engagement scale (SES)	Tomás et al., 2022
		Engagement in the classroom scale	Dierendonck et al., 2022
		Schoolwork engagement inventory (EDA)	Tomás et al., 2022
		Online student engagement questionnaire	Hoi and Hang, 2021
	Basic psychological needs	Basic psychological needs scale (BPNS)	Cromhout, 2020
			Garn et al., 2019
		Basic psychological need satisfaction and frustration scale (BPNSFS)	Tóth-Király et al., 2018
			Tóth-Király et al., 2019
		Basic psychological needs at work scale (BPNW)	Sánchez-Oliva et al., 2017
		Work-Related basic need satisfaction scale (W-BNS)	Gillet et al., 2020
		Psychological need states at work-scale (PNSW-S)	Huyghebaert-Zouaghi et al., 2021b
		Basic psychological needs in exercise scale (BPNES)	Rodrigues et al., 2021
		Psychological need states in sport-scale (PNSS-S)	Bhavsar et al., 2020
		Multidimensional perceived autonomy support scale in physical education	Burgueño et al., 2020b
		Needs-Support behaviors scale (NSBS)	Gucciardi et al., 2020
		Interpersonal supportiveness scale—coach (ISS–C)	Stenling et al., 2015
		Tripartite measure of interpersonal behaviors of coaches (TMIB-C)	Bhavsar et al., 2019
		Basic psychological need satisfaction in active commuting to and from school (BPNS-ACS)	Burgueño et al., 2020a
	Subjective task value	Subjective task value	Part et al., 2020
		Math-Related value beliefs scale	Fadda et al., 2020b
	Flow	Flow short scale (FSS)	Kyriazos et al., 2018b
		Work-Related flow inventory (WOLF)	Gu et al., 2020
	Interest	Situational interest scale	Garn, 2017
	Locus of causality	Revised perceived locus of causality (PLOC-R)	Howard et al., 2020
	Attitudes	Subjective science attitude change measures (SSACM)	Deemer et al., 2014
	Purpose	Measure of adolescent purpose (MAP)	Summers and Falco, 2020
	Self-efficacy	Work self-efficacy (W-SE)	Barbaranelli et al., 2018
		Escala de autoeficacia docente (EAD)	Dominguez-Lara et al., 2019
	Emotion regulation	Emotional processing scale (EPS-25)	Lauriola et al., 2021
		Metacognitive processes of decentering (MPoD)	Hanley et al., 2020
		Emotional responding	Clifton et al., 2020
	Happiness	Affectometer-2 (AFM-2)	Appiah et al., 2020
	Hope	Perceived hope scale (PHS)	Krafft et al., 2020
	Anxiety	Anxiety questionnaire for students (AFS)	Lohbeck and Petermann, 2019
	Fear	Death and dying anxiety inventory (FVTS)	Jastrzebski et al., 2020
	Loneliness	De Jong gierveld loneliness scale (DJGLS)	Grygiel et al., 2019
**Self and identity**			
	Self-compassion	Self-Compassion scale (SCS)	Neff et al., 2018
			Neff et al., 2019
			Tóth-Király et al., 2017
			Benda, 2018
		Self-Compassion scale—youth version (SCS-Y)	Neff et al., 2021a
		State self-compassion scale—long form (SSCS-L)	Neff et al., 2021b
	Self-concept	Academic self-concept (ASC)	Arens et al., 2021
		Strengths and difficulties questionnaire (SDQ)	Morin et al., 2016a
	Self-perception	Self-Perception profile for children (SPPC)	Arens and Morin, 2017
		Physical self-perception profile (PSPP)	Chung et al., 2016
		Early childhood parental acceptance-rejection questionnaire (ECPARQ)	Giotsa and Kyriazos, 2019
	Body checking	Body checking questionnaire (BCQ)	Maïano et al., 2021
		Body checking cognitions scale (BCCS)	Maïano et al., 2021
	Personality, big five	International personality item pool (IPIP)	Lee et al., 2017
		International personality item pool—short form (Mini-IPIP)	Arias et al., 2018
	Personality, multicultural	Multicultural personality inventory—short form (MPI-SF)	Korol et al., 2018
	Personality, psychopath	Short dark triad (SD3)	McLarnon and Tarraf, 2017
			McLarnon and Tarraf, 2021
			Vaughan et al., 2019
		Dirty dozen (DD)	McLarnon and Tarraf, 2017
		Triarchic psychopathy measure (TriPM)	Somma et al., 2019
		Machiavellian personality scale (MPS)	Gu et al., 2017
		Personality inventory for DSM-5—brief form (PID-5-BF)	Gomez et al., 2020b
	Conspiracy beliefs	Generic conspiracist belief scale (GCB)	García-Garzón et al., 2020
	Paranoid thoughts	Green et al. paranoid thoughts scale (GPTS)	Bianchi and Verkuilen, 2021
	Character strength	Encouragement character strength scale (ECSS)	Wang et al., 2021
		Values in action inventory of strengths (VIA-IS)	Ng et al., 2017
	Consideration of future consequences	Consideration of future consequences scale-14 (CFCS-12)	McKay et al., 2016
	Morningness	Composite scale of morningness (CSM)	Díaz-Morales and Parra-Robledo, 2018
			Morin et al., 2016b
	Compassion	Compassionate engagement and action scales (CEAS)	Halamová et al., 2020
		Compassion scale (CS)	Pommier et al., 2020
	Emotional intelligence	Emotional quotient inventory: youth version short (EQ-i: YV-S)	Esnaola et al., 2018
		Profile of emotional competence (PEC)	Pirsoul et al., 2022
	Intelligence	Wechsler intelligence scale for children—fifth edition (WISC–V)	Lecerf and Canivez, 2018
	Life skills	Life skills scale for sport (LSSS)	Cronin and Allen, 2017
		French psychological capital questionnaire (F-PCQ-24)	Choisay et al., 2021
		Fonctionnement optimal psychologique (FOP)	Jaotombo, 2019
	Grit	Grit-Original scale (Grit-O)	Van Zyl et al., 2020
	Mental toughness	Mental toughness inventory (MTI)	Bédard-Thom and Guay, 2018
			Schmid et al., 2018
		Mental toughness questionnaire-48 (MTQ48)	Kawabata et al., 2021
	Resilience	Characteristics of resilience in sports teams (CREST)	Decroos et al., 2017
		City tourism resilience	Dai et al., 2019
		Connor–Davidson resilience scale (CD-RISC)	Perera and Ganguly, 2018
**Depression and wellbeing**			
	Depression	Depression anxiety stress scales-21 (DASS-21)	Gomez et al., 2020a
			Jovanovic et al., 2021
			Vaughan et al., 2020
			Volmer et al., 2019
		Patient health questionnaire (PHQ-9 and PHQ-15)	Bianchi, 2020
			Cano-García et al., 2020
		Occupational depression inventory (ODI)	Bianchi and Schonfeld, 2020
		Depression indicators scale-children and adolescents (BAID-IJ)	Borges et al., 2017
		Beck depression inventory-II (BDI-II)	Høstmælingen et al., 2021
		Hamilton depression rating scale (HDRS17)	Nixon et al., 2020
	Burnout	Maslach burnout inventory—human services survey (MBI-HSS)	Doherty et al., 2021
		Burnout assessment tool (BAT)	Sakakibara et al., 2020
		Burnout and work engagement (with items of MBI and UWES)	Trógolo et al., 2020
		Athlete burnout scale (ABO-S)	Isoard-Gautheur et al., 2018
		School burnout inventory (SBI)	Tóth-Király et al., 2021
		Shirom-Melamed burnout measure (SMBM)	Bianchi, 2020
	Positive thoughts	Automatic thoughts questionnaire—positive (ATQ-P)	Appiah et al., 2020
	Wellbeing	Mental health continuum—short form (MHC-SF)	Ferentinos et al., 2019
			Lamborn et al., 2018
			Longo et al., 2020
			Reinhardt et al., 2020a
			Reinhardt et al., 2020b
			Rogoza et al., 2018
			Schutte and Wissing, 2017
			Silverman et al., 2018
		Questionnaire for eudaimonic wellbeing (QEWB)	Cromhout, 2020
			Fadda et al., 2017
			Fadda et al., 2020a
		Index of psychological wellbeing at work (IPWBW)	Morin et al., 2017
		Scales of psychological wellbeing (SPWB)	Espinoza et al., 2018
		Peer and community relational health indices (RHIP and RHIC)	Cromhout, 2020
		Scale of positive and negative experience (SPANE)	Kyriazos et al., 2018a
		Interpersonal, community, occupational, physical, psychological, economic scale (I COPPE)	Myers et al., 2016
		World health organization quality of life scale (WHOQOL-BREF)	Perera et al., 2018a
	Workaholism	Dutch work addiction scale (DUWAS)	Huyghebaert-Zouaghi et al., 2022
	Stress	University stress scale (USS)	Portoghese et al., 2020
		Détresse psychologique au travail (DPT)	Morin et al., 2016c
	Stress disorder	Posttraumatic stress disorder checklist−5 (PCL−5)	Fresno et al., 2020
	Anxiety disorder	Multidimensional social anxiety response inventory-21 (MSARI-21)	Deller et al., 2020
		State–Trait inventory of cognitive and somatic anxiety (STICSA)	Styck et al., 2021
	Problem behavior	Eyberg child behavior inventory (ECBI)	Hukkelberg, 2019
		Home and community social behavior scales (HCSBS)	Hukkelberg and Ogden, 2020
		Symptom checklist-9–revised (SCL-90-R)	Gomez et al., 2021
		Young adult version of the diagnostic interview for children (YA-DISC)	Lahey et al., 2018
	ADHD	ADHD rating scale-IV (ADHD RS-IV)	Yi et al., 2021
		ADHD questionnaire, adapted from DSM-5	Arias et al., 2016
			Frutos, 2021
		Adult ADHD self-report scale symptom checklist (ASRS)	Gomez and Stavropoulos, 2021
		Diagnostik-System für psychische störungen II (DISYPS-II)	Rodenacker et al., 2017
		Inventario de comportamiento infantil y adolescente (CABI)	Frutos, 2019
**Interpersonal relations**			
	Romantic relationships	Dyadic adjustment scale (DAS)	Vajda et al., 2019
		Relational uncertainty scale	Goodboy et al., 2021
	Parenting	Parental structure scale	Ratelle et al., 2018
	Group cohesion	Group environment questionnaire (GEQ)	Haugen et al., 2021
	Working alliance	Working alliance inventory short form (WAI-S)	Hukkelberg and Ogden, 2019
	Work teams	Role ambiguity scale (RAS)	Leo et al., 2017
	Supervision	Supervisee levels questionnaire (SLQ-R)	Junga et al., 2019
	Perceived social support	Social provisions scale (SPS)	Perera, 2016
**Other**			
	Motoric skills	Test of gross motor development-third edition (TGMD-3)	Garn and Webster, 2021
	Scar evaluation	Patient-Reported scar evaluation questionnaire (PR-SEQ)	Sen et al., 2015

The first domain of *learning and instruction* includes the constructs of cooperative learning (Fernandez-Rio et al., 2022) and instructional quality (Scherer et al., 2016).

The second domain of *motivation and emotion* includes the constructs academic motivation (Litalien et al., 2017; Guay and Bureau, 2018; Gordeeva et al., 2020; Howard et al., 2020; Kartal, 2020; Guay et al., 2021; Dierendonck et al., 2022), sport motivation (Appleton et al., 2016; Milton et al., 2018; Stenling et al., 2018; Cece et al., 2019; Méndez-Giménez et al., 2020; Rodrigues et al., 2020), work motivation (Burk and Wiese, 2018; Calkins, 2018; Howard et al., 2018, 2020, 2021; Gegenfurtner and Quesada-Pallarès, 2022), job satisfaction (Sutherland, 2020), affective commitment (Perreira et al., 2018), work engagement (Gillet et al., 2019; Huyghebaert-Zouaghi et al., 2021a, 2022), teacher engagement (Perera et al., 2018b), student engagement (Hoi and Hang, 2021; Dierendonck et al., 2022; Tomás et al., 2022), basic psychological needs (Stenling et al., 2015; Sánchez-Oliva et al., 2017; Tóth-Király et al., 2018, 2019; Bhavsar et al., 2019, 2020; Garn et al., 2019; Burgueño et al., 2020a,b; Cromhout, 2020; Gillet et al., 2020; Gucciardi et al., 2020; Huyghebaert-Zouaghi et al., 2021b; Rodrigues et al., 2021), subjective task value (Fadda et al., 2020b; Part et al., 2020), flow (Kyriazos et al., 2018b; Gu et al., 2020), interest (Garn, 2017), locus of causality (Howard et al., 2020), attitudes (Deemer et al., 2014), purpose (Summers and Falco, 2020), self-efficacy (Barbaranelli et al., 2018; Dominguez-Lara et al., 2019), emotion regulation (Clifton et al., 2020; Hanley et al., 2020; Lauriola et al., 2021), happiness (Appiah et al., 2020), hope (Krafft et al., 2020), anxiety (Lohbeck and Petermann, 2019), fear (Jastrzebski et al., 2020), and loneliness (Grygiel et al., 2019).

The third domain of *self and identity* includes the constructs of self-compassion (Tóth-Király et al., 2017; Benda, 2018; Neff et al., 2018, 2019, 2021a,b), self-concept (Morin et al., 2016a; Arens et al., 2021), self-perception (Chung et al., 2016; Arens and Morin, 2017; Giotsa and Kyriazos, 2019), body checking (Maïano et al., 2021), big five personality traits (Lee et al., 2017; Arias et al., 2018), multicultural personality (Korol et al., 2018), psychopath personality (Gu et al., 2017; McLarnon and Tarraf, 2017, 2021; Somma et al., 2019; Gomez et al., 2020b; Vaughan et al., 2020), conspiracy beliefs (García-Garzón et al., 2020), paranoid thoughts (Bianchi and Verkuilen, 2021), character strength (Ng et al., 2017; Wang et al., 2021), consideration of future consequences (McKay et al., 2016), morningness (Morin et al., 2016b; Díaz-Morales and Parra-Robledo, 2018), compassion (Halamová et al., 2020; Pommier et al., 2020), emotional intelligence (Esnaola et al., 2018; Pirsoul et al., 2022), intelligence (Lecerf and Canivez, 2018), life skills (Cronin and Allen, 2017; Jaotombo, 2019; Choisay et al., 2021), mental toughness (Bédard-Thom and Guay, 2018; Schmid et al., 2018; Kawabata et al., 2021), and resilience (Decroos et al., 2017; Perera and Ganguly, 2018; Dai et al., 2019).

The fourth dimension of *depression and wellbeing* includes the constructs of depression (Borges et al., 2017; Volmer et al., 2019; Bianchi, 2020; Bianchi and Schonfeld, 2020; Cano-García et al., 2020; Gomez et al., 2020a; Nixon et al., 2020; Vaughan et al., 2020; Høstmælingen et al., 2021; Jovanovic et al., 2021), burnout (Isoard-Gautheur et al., 2018; Bianchi, 2020; Sakakibara et al., 2020; Trógolo et al., 2020; Doherty et al., 2021; Tóth-Király et al., 2021), positive thoughts (Appiah et al., 2020), wellbeing (Myers et al., 2016; Fadda et al., 2017, 2020a; Morin et al., 2017; Schutte and Wissing, 2017; Espinoza et al., 2018; Kyriazos et al., 2018a; Lamborn et al., 2018; Perera et al., 2018a; Rogoza et al., 2018; Silverman et al., 2018; Ferentinos et al., 2019; Cromhout, 2020; Longo et al., 2020; Reinhardt et al., 2020a,b), workaholism (Huyghebaert-Zouaghi et al., 2022), stress (Morin et al., 2016c; Portoghese et al., 2020), stress disorder (Fresno et al., 2020), anxiety disorder (Deller et al., 2020; Styck et al., 2021), problem behavior (Lahey et al., 2018; Hukkelberg, 2019; Hukkelberg and Ogden, 2020; Gomez et al., 2021), and attention deficit hyperactivity disorder (Arias et al., 2016; Rodenacker et al., 2017; Frutos, 2019, 2021; Gomez and Stavropoulos, 2021; Yi et al., 2021).

The fifth dimension of *interpersonal relations* includes the constructs of romantic relationships (Vajda et al., 2019; Goodboy et al., 2021), parenting (Ratelle et al., 2018), group cohesion (Haugen et al., 2021), working alliance (Hukkelberg and Ogden, 2019), work teams (Leo et al., 2017), supervision (Junga et al., 2019), and perceived social support (Perera, 2016).

The sixth and last category includes two constructs that did not fit the aforementioned five domains: motoric skills (Garn and Webster, 2021) and scar evaluation (Sen et al., 2015).

The 158 included studies psychometrically tested a total of 135 different scales. Among the most often-investigated constructs are wellbeing (*n* = 17), basic psychological needs (*n* = 14 studies), depression (*n* = 10), academic motivation (*n* = 7), psychopathic personality (*n* = 7), sport motivation (*n* = 7), attention deficit hyperactivity disorder (*n* = 6), burnout (*n* = 6), self-compassion (*n* = 6), and work motivation (*n* = 6). Scales whose factorial structures were frequently examined using a B-ESEM approach include the Mental Health Continuum—Short Form (MHC-SF), addressed in eight studies, the Academic Motivation Scale (AMS), addressed in six studies, and the Self-Compassion Scale (SCS), addressed in six studies.

### Research question 2: Model fit

**Figure 3:**
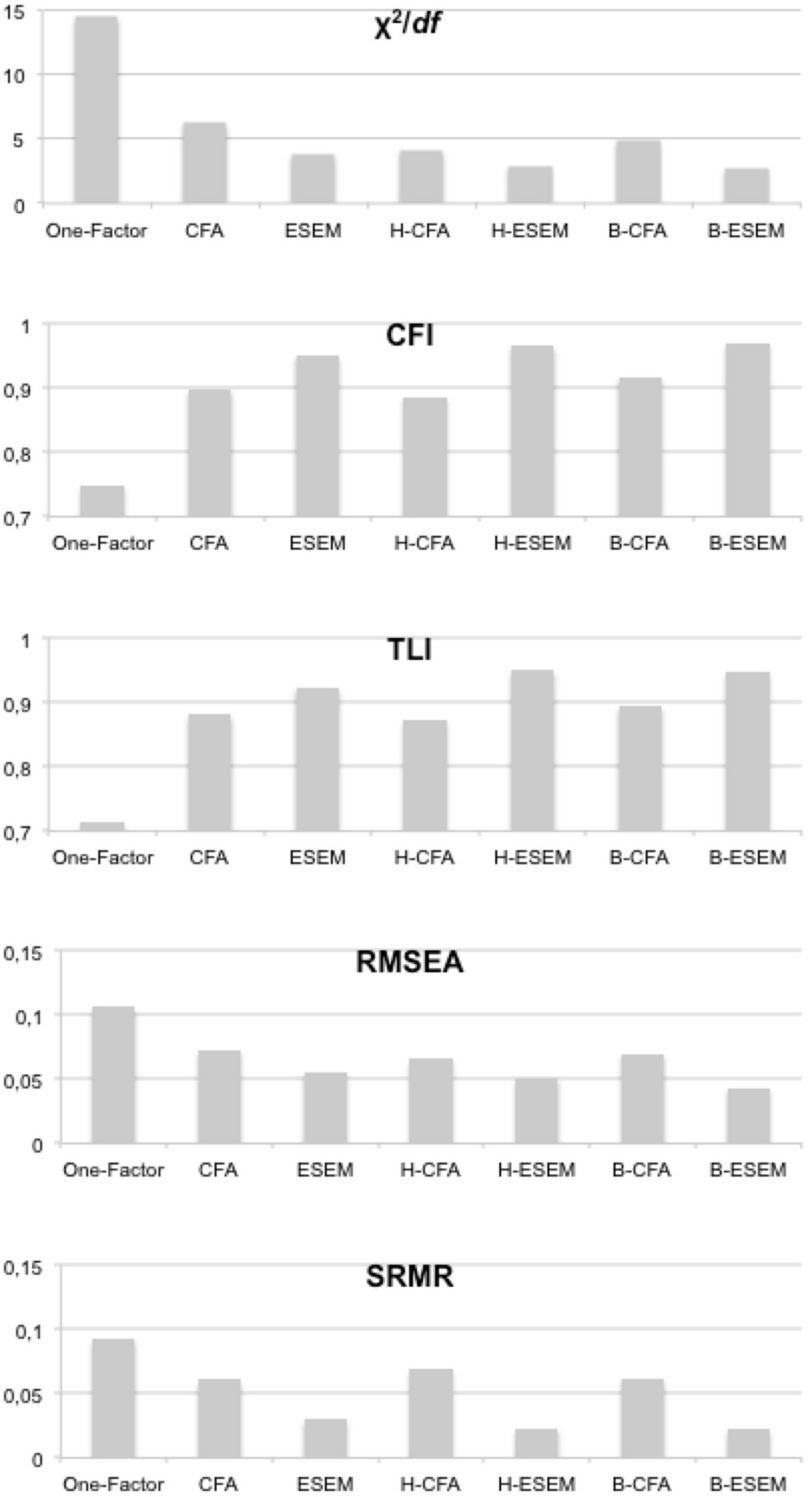
Goodness-of-fit indices per model representation.

Research question 2 asked, “What is the model fit of B-ESEM representations in comparison to CFA, ESEM, H-CFA, H-ESEM, and B-CFA representations?” To answer this research question, a primary meta-analysis was performed to cumulate the reported goodness-of-fit-indices across studies and then to generate average estimates of model fit for each representation. Table 6 reports the results of this meta-analysis. Analyses of variance suggest that model fit differed significantly between representations. The findings of comparing the model representations, as shown in Figure 1, indicate that the B-ESEM representation showed the best model fit, with the lowest χ^2^*/df* ratio, the highest CFI, the lowest RMSEA, and the lowest SRMR. TLI was minimally higher for H-ESEM (0.950) than for B-ESEM (0.948) [ΔTLI = 0.002, *t*_(23)_ = 1.321, *p* = 0.200]. These results demonstrate the superior model fit of B-ESEM solutions over other factorial representations across a range of scales and domains. Figure 3 portrays the strength of the B-ESEM goodness-of-fit indices relative to the other model representations.

**Table 6:** Mean (and standard deviation) estimates of the goodness-of-fit indices per model.

	***χ^2^/df***	**CFI**	**TLI**	**RMSEA**	**SRMR**
One-factor	14.506 (18.531)	0.748 (0.161)	0.715 (0.174)	0.106 (0.040)	0.093 (0.043)
CFA	6.339 (10.551)	0.898 (0.092)	0.881 (0.095)	0.073 (0.033)	0.061 (0.041)
ESEM	3.869 (3.665)	0.952 (0.051)	0.924 (0.085)	0.056 (0.037)	0.030 (0.014)
H-CFA	4.190 (2.560)	0.886 (0.071)	0.872 (0.077)	0.066 (0.028)	0.069 (0.029)
H-ESEM	2.940 (1.513)	0.966 (0.020)	0.950 (0.027)	0.051 (0.026)	0.023 (0.006)
B-CFA	4.891 (3.882)	0.918 (0.071)	0.895 (0.080)	0.070 (0.030)	0.062 (0.079)
B-ESEM	2.697 (1.929)	0.971 (0.035)	0.948 (0.056)	0.043 (0.017)	0.022 (0.012)
*F*_(1, 6)_	33.563	131.653	96.665	69.902	29.240
*p*	< 0.001	< 0.001	< 0.001	< 0.001	< 0.001
*Partial* η*^2^*	0.848	0.956	0.942	0.921	0.830
90% CI	0.442–0.907	0.803–0.973	0.744–0.963	0.666–0.951	0.396–0.896

CFI, comparative fit index; TLI, Tucker-Lewis index, RMSEA, root mean square error of approximation, SRMR, standardized root mean squared residual. CFA, confirmatory factor analysis; ESEM, exploratory structural equation modeling; H-CFA, hierarchical confirmatory factor analysis; H-ESEM, hierarchical exploratory structural equation modeling; B-CFA, bifactor confirmatory factor analysis; B-ESEM, bifactor structural equation modeling.

Table 7 presents a correlation matrix of the goodness-of-fit indices of all model representations. Across models, correlations were highly positive between CFI and TLI. Correlations also tended to be substantial between the same fit indices of different models, for example between ESEM RMSEA, H-ESEM RMSEA, and B-ESEM RMSEA or between one-factor χ^2^*/df*, CFA χ^2^*/df*, and ESEM χ^2^*/df* . Correlation coefficients between the incremental (CFI, TLI) and absolute (RMSEA, SRMR) fit indices tended to be negative.

**Table 7:** Correlation matrix.

	**Variable**	**01**	**02**	**03**	**04**	**05**	**06**	**07**	**08**	**09**	**10**	**11**	**12**	**13**	**14**	**15**	**16**
01	Sample size	–															
02	Item number	0.27	–														
03	Specific factor number	0.37	0.69	–													
04	General factor number	−0.01	0.10	0.18	–												
05	One-factor *χ^2^/df*	0.81	−0.21	−0.12	0.08	–											
06	One-factor CFI	0.06	0.14	0.05	−0.34	0.05	–										
07	One-factor TLI	0.06	0.19	0.10	−0.32	0.05	0.97	–									
08	One-factor RMSEA	0.09	−0.47	−0.32	0.16	0.38	−0.25	−0.27	–								
09	One-factor SRMR	−0.08	0.25	0.15	0.25	0.15	−0.74	−0.75	0.56	–							
10	CFA *χ^2^/df*	0.38	−0.08	0.03	0.03	0.89	0.09	0.10	0.20	−0.03	–						
11	CFA CFI	−0.03	−0.16	0.02	−0.13	0.23	0.71	0.70	0.14	−0.30	−0.02	–					
12	CFA TLI	−0.02	−0.12	0.05	−0.13	0.24	0.71	0.71	0.13	−0.30	−0.02	0.96	–				
13	CFA RMSEA	−0.09	−0.20	−0.19	0.00	0.06	−0.19	−0.20	0.56	0.32	0.54	−0.44	−0.32	–			
14	CFA SRMR	−0.04	0.02	−0.13	0.00	−0.17	−0.15	−0.71	−0.03	0.16	0.00	−0.31	−0.75	0.24	–		
15	ESEM *χ^2^/df*	0.66	−0.18	−0.24	−0.01	0.72	0.05	0.04	0.23	−0.05	0.48	0.00	−0.01	0.21	−0.05	–	
16	ESEM CFI	−0.06	−0.07	0.12	−0.08	0.11	0.51	0.49	0.06	−0.34	−0.02	0.73	0.71	−0.17	−0.29	−0.17	–
17	ESEM TLI	−0.02	−0.03	0.06	−0.07	0.10	0.57	0.56	0.03	−0.36	−0.03	0.54	0.53	−0.13	−0.71	−0.17	0.71
18	ESEM RMSEA	−0.05	−0.18	−0.19	−0.02	0.11	−0.10	−0.12	0.61	0.28	0.03	−0.05	−0.06	0.33	0.26	17	−0.20
19	ESEM SRMR	−0.01	−0.07	−0.34	−0.09	−0.23	−0.31	−0.33	−0.18	0.17	−0.10	−0.68	−0.69	0.33	0.37	0.11	−0.83
20	H-CFA *χ^2^/df*	0.66	−0.35	−0.12	−0.16	0.76	−0.16	−0.17	0.38	0.00	0.75	0.11	0.06	0.39	−0.35	0.64	0.23
21	H-CFA CFI	−0.18	−0.07	−0.28	−0.33	−0.37	0.84	0.84	−0.15	−0.43	−0.10	0.68	0.69	−0.13	−0.73	−0.14	0.78
22	H-CFA TLI	−0.15	−0.02	−0.25	−0.32	−0.39	0.87	0.85	−0.18	−0.42	−0.08	0.64	0.67	−0.14	−0.73	−0.14	0.75
23	H-CFA RMSEA	−0.13	−0.46	−0.28	0.02	0.18	−0.23	−0.25	0.44	−0.25	0.44	0.00	−0.03	0.90	0.45	0.24	0.04
24	H-CFA SRMR	−0.27	−0.03	−0.03	0.04	−0.15	−0.66	−0.66	−0.44	−0.02	−0.15	−0.26	−0.45	0.26	0.50	−0.03	−0.49
25	H-ESEM *χ^2^/df*	0.16	−0.46	−0.42	–	0.38	0.06	0.04	0.21	−0.68	0.41	−0.07	−0.12	0.18	−0.65	0.93	−0.44
26	H-ESEM CFI	0.09	0.12	0.25	–	−0.08	0.66	0.73	−0.44	−0.04	0.13	0.71	0.60	0.12	−0.42	−0.43	0.96
27	H-ESEM TLI	0.09	0.20	0.33	–	−0.18	0.71	0.71	−0.56	0.09	0.11	0.67	0.62	0.06	−0.27	−0.48	0.96
28	H-ESEM RMSEA	−0.32	−0.52	−0.49	–	0.10	0.05	0.02	0.44	−0.51	0.35	−0.14	−0.24	0.79	−0.27	0.69	−0.15
29	H-ESEM SRMR	−0.93	0.49	−0.41	–	−0.89	0.01	0.04	0.16	0.31	−0.86	−0.42	−0.45	0.72	0.56	0.01	−0.75
30	B-CFA *χ^2^/df*	0.64	−0.19	−0.06	0.01	0.79	0.05	0.06	0.21	−0.10	0.80	0.03	0.03	0.27	−0.11	0.79	0.04
31	B–CFA CFI	0.15	−0.29	−0.28	−0.05	0.28	0.72	0.71	0.20	−0.21	0.11	0.64	0.61	−0.10	−0.09	0.08	0.39
32	B-CFA TLI	0.17	−0.22	−0.25	−0.04	0.30	0.74	0.73	0.17	−0.21	0.11	0.61	0.60	−0.14	−0.41	0.05	0.36
33	B-CFA RMSEA	−0.26	−0.18	−0.06	−0.07	−0.06	−0.14	−0.15	0.62	0.16	0.08	0.00	0.01	0.70	−0.09	0.05	0.10
34	B-CFA SRMR	−0.17	0.19	0.29	−0.03	−0.46	−0.41	−0.39	−0.30	−0.04	−0.20	−0.34	−0.01	−0.13	−0.03	−0.19	−0.02
35	B-ESEM *χ^2^/df*	0.67	−0.16	−0.20	−0.02	0.66	−0.03	−0.04	0.20	0.07	0.42	−0.02	−0.02	0.15	−0.03	0.85	−0.18
36	B-ESEM CFI	−0.08	−0.15	0.00	−0.08	0.23	0.53	0.51	0.13	−0.37	0.08	0.64	0.66	−0.06	−0.27	−0.02	0.88
37	B-ESEM TLI	−0.04	−0.08	0.02	−0.09	0.23	0.58	0.56	0.09	−0.35	0.11	0.58	0.57	−0.07	−0.46	0.00	0.74
38	B-ESEM RMSEA	−0.06	−0.13	−0.18	0.00	−0.12	−0.28	−0.30	0.37	0.10	0.01	−0.25	−0.22	0.48	0.26	0.19	−0.44
39	B-ESEM SRMR	0.04	0.16	−0.17	−0.03	−0.44	−0.39	−0.43	−0.37	−0.24	−0.24	−0.54	−0.54	0.12	0.31	−0.12	−0.53
Two-tailed Pearson correlation coefficients.																	
		**17**	**18**	**19**	**20**	**21**	**22**	**23**	**24**	**25**	**26**	**27**	**28**	**29**	**30**	**31**	**32**
17	ESEM TLI	-															
18	ESEM RMSEA	−0.16	−														
19	ESEM SRMR	−0.79	0.59	−													
20	H-CFA χ^2^/*df*	0.20	0.08	−0.58	−												
21	H-CFA CFI	0.77	−0.15	−0.55	−0.05	−											
22	H-CFA TLI	0.76	−0.18	−0.57	−0.08	0.99	−										
23	H-CFA RMSEA	−0.05	0.79	0.33	0.40	−0.17	−0.22	−									
24	H-CFA SRMR	−0.52	0.38	0.50	−0.20	−0.40	−0.57	0.58	−								
25	H-ESEM χ^2^/*df*	−0.61	0.57	−0.05	0.43	−0.08	−0.10	0.27	−0.70	−							
26	H-ESEM CFI	0.87	−0.16	−0.74	−0.01	0.89	0.84	0.03	−0.63	−0.35	−						
27	H-ESEM TLI	0.93	−0.26	−0.83	0.00	0.83	0.82	−0.12	−0.30	−0.45	0.98	−					
28	H-ESEM RMSEA	−0.41	0.98	0.50	0.43	−0.13	−0.25	−0.81	−0.32	0.68	−0.17	−0.29	−				
29	H-ESEM SRMR	0.70	0.64	0.99	−0.97	−0.42	−0.70	0.82	0.61	−0.08	−0.75	−0.82	0.46	−			
30	B-CFA χ^2^/*df*	0.03	0.02	−0.15	0.83	−0.22	−0.20	0.45	−0.24	0.38	0.11	0.14	0.34	−0.96	−		
31	B-CFA CFI	0.38	0.06	−0.42	0.16	0.61	0.57	0.07	−0.53	0.04	0.81	0.76	0.09	−0.43	−0.01	−	
32	B-CFA TLI	0.38	0.04	−0.43	0.11	0.61	0.59	0.00	0.90	−0.11	0.54	0.59	−0.25	−0.88	−0.03	0.98	−
33	B-CFA RMSEA	−0.02	0.21	0.11	0.39	−0.21	−0.24	0.91	0.44	0.27	0.05	−0.04	0.80	0.95	0.35	−0.38	−0.42
34	B-CFA SRMR	−0.03	−0.10	−0.04	−0.39	−0.59	−0.59	0.63	0.61	0.40	−0.77	−0.95	0.78	0.83	−0.11	−0.46	−0.47
35	B-ESEM χ^2^/*df*	−0.18	0.11	0.08	0.59	−0.25	−0.24	0.26	−0.12	0.89	−0.55	−0.61	0.70	0.29	0.70	0.07	0.06
36	B-ESEM CFI	0.62	−0.10	−0.67	0.25	0.74	0.69	0.09	−0.10	−0.13	0.86	−0.86	−0.07	−0.83	0.12	0.43	0.39
37	B-ESEM TLI	0.55	−0.10	−0.52	0.21	0.75	0.73	0.01	−0.21	−0.28	0.77	0.83	−0.24	−0.85	0.13	0.40	0.39
38	B-ESEM RMSEA	−0.36	0.35	0.46	0.07	−0.28	−0.29	0.75	0.28	0.50	−0.24	−0.32	0.93	0.78	0.02	−0.11	−0.15
39	B-ESEM SRMR	−0.49	0.25	0.66	−0.53	−0.68	−0.64	0.22	0.22	−0.35	−0.77	−0.74	0.16	0.92	−0.19	−0.53	−0.53
		**33**	**34**	**35**	**36**	**37**	**38**	**39**									
33	B-CFA RMSEA	−															
34	B-CFA SRMR	0.21	−														
35	B-ESEM χ^2^/*df*	0.00	−0.20	−													
36	B-ESEM CFI	0.08	−0.03	−0.11	−												
37	B-ESEM TLI	0.09	−0.01	−0.11	0.85	−											
38	B-ESEM RMSEA	0.44	−0.04	0.33	−0.47	−0.52	−										
39	B-ESEM SRMR	0.32	0.05	−0.05	−0.56	−0.47	0.39	−									

### Research question 3: Influence of sample size, item number, and factor number on model fit

Research question 3 asked, “How sensitive is model fit to sample size, item number, and the number of specific and general factors in a model?” To answer this research question, we performed within-study and between-study analyses.

First, within-study analyses aggregated the change in model fit as a result of changes in B-ESEM model structure. A number of studies reported model fit for different model specifications. For example, Yi et al. (2021) examined the fit of a B-ESEM model with two or three specific factors; their findings demonstrate better model fit for a three-factor representation. A variety of different representations were explored among the included studies, such as one and three general factors (Bhavsar et al., 2019), as well as three and nine specific factors (Bhavsar et al., 2019), four and five specific factors (Choisay et al., 2021), four and six specific factors (Part et al., 2020), seven and eight specific factors (Bédard-Thom and Guay, 2018), and four and twenty specific factors (Sutherland, 2020). Table 8 presents within-study analyses of changes in B-ESEM model fit by factor number. Average estimates across reports suggest that, when the number of specific factors changed from two to three, two to six, three to four, or three to six, CFI and TLI increased, while the χ^2^*/df* ratio, RMSEA, and SRMR decreased. Similarly, when the number of general factors changed from one to two, CFI and TLI increased, while the χ^2^*/df* ratio, RMSEA, and SRMR decreased.

**Table 8:** Within-Study analysis of changes in B-ESEM model fit by factor number.

**Factor change**	**Study**	**Δ *χ^2^/df***	**Δ CFI**	**Δ TLI**	**Δ RMSEA**	**Δ SRMR**
2–3 s	Arias et al. (2016)	−0.978	0.016	0.022	−0.016	–
	Frutos (2019)	−1.349	0.008	0.009	−0.009	–
	Frutos (2021)	–	0.009	0.012	−0.016	–
	Giotsa and Kyriazos (2019)	−0.257	0.020	0.022	−0.005	−0.005
	Giotsa and Kyriazos (2019)	−0.467	0.023	0.027	−0.007	−0.007
	Gomez and Stavropoulos (2021)	0.131	0.003	−0.005	0.002	–
	Gu et al. (2020)	−3.458	0.050	0.080	−0.040	–
	Nixon et al. (2020)	−0.069	0.040	0.045	−0.008	–
	Rodenacker et al. (2017)	−0.865	0.004	–	−0.006	–
	Rodenacker et al. (2017)	−0.600	0.003	–	−0.005	–
	Tóth-Király et al. (2018)	−4.647	0.057	0.070	−0.018	–
	Tóth-Király et al. (2018)	−1.067	0.052	0.065	−0.022	–
	Yi et al. (2021)	−0.626	0.008	0.011	−0.017	–
	Average	−1.188	0.023	0.032	−0.013	−0.006
2–6 s	Rodrigues et al. (2020)	−1.235	0.088	0.077	−0.019	−0.021
	Rodrigues et al. (2020)	−1.650	0.098	0.107	−0.024	−0.024
	Tóth-Király et al. (2018)	−8.410	0.098	0.127	−0.044	–
	Tóth-Király et al. (2018)	−1.564	0.074	0.095	−0.041	–
	Average	−3.215	0.090	0.102	−0.032	−0.023
3–4 s	Cromhout (2020)	−0.089	0.016	0.019	−0.005	−0.006
	Cromhout (2020)	0.038	0.000	−0.005	0.002	−0.003
	Cromhout (2020)	−0.284	0.024	0.082	−0.025	−0.008
	Cromhout (2020)	0.276	−0.010	−0.050	0.007	−0.004
	Fadda et al. (2017)	−0.045	0.010	0.006	−0.001	–
	Giotsa and Kyriazos (2019)	−0.080	0.006	0.004	−0.002	−0.003
	Sutherland (2020)	−0.006	0.002	0.001	0.000	–
	Average	−0.027	0.007	0.008	−0.003	−0.005
3–6 s	Espinoza et al. (2018)	−0.583	0.086	0.086	−0.012	**–**
	Espinoza et al. (2018)	−0.597	0.101	0.099	−0.013	**–**
	Tóth-Király et al. (2018)	−3.763	0.041	0.057	−0.026	**–**
	Tóth-Király et al. (2018)	−2.233	0.025	0.033	−0.017	**–**
	Tóth-Király et al. (2018)	−0.498	0.022	0.030	−0.019	**–**
	Average	−1.535	0.055	0.061	−0.017	**–**
1–2 g	Neff et al. (2018)	−0.396	0.000	0.010	−0.010	–
	Rodrigues et al. (2020)	−0.690	0.017	0.017	−0.005	−0.005
	Rodrigues et al. (2020)	−0.314	0.014	0.020	−0.007	−0.005
	Tóth-Király et al. (2018)	−0.093	0.000	−0.002	0.001	–
	Tóth-Király et al. (2018)	−1.437	0.016	0.022	−0.008	–
	Tóth-Király et al. (2018)	−0.243	0.010	0.015	−0.008	–
	Tóth-Király et al. (2019)	0.017	0.000	−0.001	0.000	–
	Average	−0.451	0.008	0.012	−0.005	−0.005

2–3 s, change from two specific to three specific factors; 2–6 s, change from two specific to six specific factors; 3–4 s, change from three specific to four specific factors; 3–6 s, change from three specific to six specific factors; 1–2 g, change from one general factor to two general factors.

Second, between-study analyses were performed to estimate the extent to which model fit is influenced by sample size, item number, and the number of specific and general factors. Between-study analyses included correlation analyses and meta-regression analysis. The correlation analysis shown in Table 7 suggests high correlation coefficients between sample size and the χ^2^/*df* ratio. Prior to the meta-regression, an outlier detection analysis was conducted to identify studies with outlying values. Analyses were performed separately for sample size, item number, number of specific factors, and number of general factors. Table 9 presents the outcomes of the outlier analysis. For sample size, 40 reports with more than 1,763 participants were identified. For item number, 12 reports examined scales with 44 items or more. For the number of specific factors, 19 reports used B-ESEM representations with nine specific factors or more. For the number of general factors, 13 reports used B-ESEM representations with more than one general factor. These reports were removed prior to the meta-regression analysis.

**Table 9:** Outlier detection analysis.

**Variable**	***Md***	***MAD***	**Interval**	***N***
Sample size	573	396.596	−616.787 < *x_*i*_* < 1,762.787	40
Item number	21	7.413	−1.239 < *x_*i*_* < 43.239	12
Number of specific factor	4	1.483	−0.449 < *x_*i*_* < 8.449	19
Number of general factors	1	0	1 < *x_*i*_* < 1	13

Md, median; MAD, median absolute deviation; N, number of detected outliers.

Table 10 presents the results of the meta-regression. First, the findings indicate that sample size significantly influenced the χ^2^*/df* ratio in all models except H-ESEM. This finding supports simulation study results on the sample-size sensitivity of the χ^2^*/df* ratio. Second, item number influenced (a) CFI and TLI in all models except ESEM, (b) RMSEA in CFA, H-CFA, and H-ESEM, and (c) SRMR in all models except ESEM and H-ESEM. Third, the number of specific factors influenced (a) CFI and TLI in all models except CFA, H-ESEM, and B-ESEM, (b) RMSEA in the CFA, ESEM, H-ESEM, B-CFA, and B-ESEM representations, and (c) SRMR in the ESEM, H-CFA, B-CFA, and B-ESEM representations.

**Table 10:** Between-Study analysis of the influence of sample size, item number, and specific factor number on model fit.

	**Sample size**				**Item number**				**Number of specific factors**			
**Model**	***n***	**β**	***SE***	***p***	***n***	**β**	***SE***	***p***	***n***	**β**	***SE***	***p***
**One-factor**
*χ^2^/df*	98	0.621	0.080	0.001***	112	−0.022	0.095	0.820	115	0.026	0.094	0.781
CFI	98	−0.126	0.101	0.212	112	−0.492	0.083	0.001***	115	−0.260	0.090	0.005**
TLI	91	−0.144	0.104	0.171	105	−0.491	0.085	0.001***	108	−0.222	0.094	0.021*
RMSEA	97	0.192	0.100	0.058^†^	111	0.013	0.095	0.889	114	0.120	0.093	0.201
SRMR	50	0.266	0.138	0.060^†^	59	0.357	0.123	0.005**	59	0.172	0.129	0.188
**CFA**
*χ^2^/df*	202	0.397	0.065	0.001***	226	−0.041	0.067	0.541	230	−0.048	0.066	0.471
CFI	228	0.127	0.066	0.055^†^	251	−0.187	0.062	0.003**	255	0.093	0.062	0.137
TLI	212	0.119	0.068	0.083^†^	235	−0.160	0.065	0.014*	239	0.123	0.064	0.056^†^
RMSEA	227	−0.086	0.066	0.194	251	−0.124	0.063	0.049*	255	−0.141	0.062	0.024*
SRMR	93	−0.076	0.104	0.468	107	0.200	0.095	0.038*	107	−0.125	0.096	0.198
**ESEM**
*χ^2^/df*	204	0.560	0.058	0.001***	225	−0.095	0.067	0.154	225	−0.122	0.066	0.067^†^
CFI	228	0.061	0.066	0.361	249	−0.078	0.063	0.218	249	0.251	0.061	0.001***
TLI	217	0.010	0.068	0.883	238	−0.050	0.065	0.441	238	0.114	0.065	0.080^†^
RMSEA	227	−0.018	0.067	0.786	249	−0.093	0.063	0.142	249	−0.172	0.063	0.007**
SRMR	94	−0.152	0.102	0.142	109	−0.007	0.096	0.938	105	−0.523	0.084	0.001***
**H-CFA**
*χ^2^/df*	47	0.540	0.124	0.001***	46	−0.094	0.148	0.531	46	0.055	0.149	0.711
CFI	48	−0.027	0.146	0.854	47	−0.396	0.135	0.005**	47	−0.295	0.141	0.042*
TLI	45	−0.069	0.150	0.650	44	−0.486	0.133	0.001***	44	−0.358	0.142	0.016*
RMSEA	48	−0.143	0.144	0.325	47	−0.292	0.141	0.044*	47	−0.114	0.146	0.439
SRMR	23	0.026	0.213	0.906	24	0.843	0.112	0.001***	22	0.519	0.187	0.011*
**H-ESEM**
*χ^2^/df*	20	0.116	0.228	0.617	21	−0.313	0.212	0.157	21	−0.137	0.222	0.544
CFI	20	−0.578	0.187	0.006**	21	−0.488	0.195	0.021*	21	−0.122	0.222	0.588
TLI	19	−0.532	0.200	0.016*	20	−0.381	0.212	0.088^†^	20	−0.021	0.229	0.928
RMSEA	20	−0.373	0.213	0.096^†^	21	−0.505	0.193	0.017*	21	−0.445	0.200	0.038*
SRMR	5	−0.298	0.477	0.567	7	0.440	0.367	0.276	7	−0.438	0.367	0.278
**B-CFA**
*χ^2^/df*	168	0.443	0.069	0.001***	187	−0.143	0.073	0.050^†^	192	0.093	0.072	0.197
CFI	189	0.012	0.073	0.871	208	−0.455	0.062	0.001***	213	−0.332	0.065	0.001***
TLI	176	−0.026	0.076	0.728	195	−0.408	0.066	0.001***	200	−0.326	0.067	0.001***
RMSEA	188	−0.177	0.072	0.015*	207	−0.012	0.070	0.869	212	0.213	0.067	0.002**
SRMR	67	−0.125	0.122	0.311	80	0.220	0.110	0.049*	81	0.220	0.109	0.047*
**B–ESEM**
*χ^2^/df*	238	0.454	0.058	0.001***	258	0.046	0.062	0.458	258	−0.052	0.062	0.408
CFI	267	0.128	0.061	0.036*	287	−0.220	0.058	0.001***	287	0.095	0.059	0.108
TLI	247	0.134	0.063	0.034*	267	−0.300	0.058	0.001***	267	0.025	0.061	0.689
RMSEA	265	−0.048	0.061	0.435	286	0.064	0.059	0.282	286	−0.122	0.059	0.040*
SRMR	104	−0.171	0.097	0.082^†^	119	0.183	0.090	0.045*	115	−0.298	0.089	0.001***

n, number of studies; β, standardized beta coefficients; SE, standard error.

^†^p < 0.10, ^*^p < 0.05, ^**^p < 0.01, ^***^p < 0.001.

As a cautionary note, goodness-of-fit is a critical component in evaluating support for a model, but it is not the only one. Particularly when fitting a more parsimonious a priori model with a less parsimonious ex-post-facto model, there is danger in simply comparing fit. Similarly, set-ESEM (Marsh et al., 2020) allows for greater parsimony by keeping theoretically independent dimensions from having cross-loadings. There needs to be some focus on why the bi-factor model is theoretically and substantively more relevant as well as providing a better fit. A saturated model, for example, will necessarily fit better than any of the models, but it would provide an appropriate test of the underlying theoretical model or a substantively relevant model. If the theoretical model is a CFA or ESEM factor model, then strong support for a Bi-ESEM reflects a failure of the a priori prediction. From this perspective, it is important to classify results in relation to the a priori model. Similarly, when comparing nested and non-nested models, the less parsimonious model necessarily fits better for models that do not take into account parsimony. Hence, the size of the difference is relevant, but not necessarily the direction. More interesting are comparisons between non-nested models. These cautionary notes can be useful to avoid over-interpreting goodness-of-fit: even if a Bi-ESEM shows superior fit, it is not necessarily the preferred model which is contingent on theoretical considerations, particularly when bifactor modeling was applied to a single-level rather than a two-level sampling approach (Eid et al., 2017).

## Conclusion

This systematic review and meta-analysis aimed to collect and describe studies using a B-ESEM framework in multivariate behavioral research, aggregate their reported model fit, and analyze model fit differences in comparison to reference models and as a function of sample size, item number, and factor number.

This meta-analysis is the first to replicate findings from simulation studies with real data on the superiority of B-ESEM models and examine the relative influence of sample size, item number, and factor number on model fit (Hu and Bentler, 1999; Marsh et al., 2004; Shi et al., 2019). While meta-analyses of structural equation models have been performed (MASEM; e.g., Cheung and Chan, 2005; Reinhold et al., 2018), this meta-analysis is also among the first to synthesize B-ESEM model fit and compare fit between models meta-analytically. This meta-analysis also documented how widespread B-ESEM has been used within the past 6 years since the seminal paper of Morin et al. (2016a) has been published: B-ESEM is now used in multiple domains to explore the multidimensional structure of numerous constructs in educational and psychological research.

Because the review included original empirical reports only—and excluded simulation studies—our examination of the influence of item number and factor number was contingent on and limited to what had been published in original empirical research. Another limitation concerned the domains within which B-ESEM has been applied. Although research in the domains of learning and instruction, motivation and emotion, self and identity, depression and wellbeing, and interpersonal relations covers large areas of educational and psychological research, the findings of this meta-analytic review are limited to these fields and cannot easily be generalized to other domains within which B-ESEM may be applied in the future. Still, we are confident that the present research synthesis—focusing on five fit indices and six reference models, cumulating 158 studies with 308 reports of B-ESEM model fit from a total sample size of 778,624 participants, and examining the relative influence of sample size, item number, and factor number within and between studies—can inform further applications of B-ESEM to identify construct-relevant psychometric multidimensionality. Future research is encouraged to examine how particular constructs and scales in educational and psychological research can be represented with B-ESEM (Morin et al., 2016a).

## Data availability statement

The raw data supporting the conclusions of this article will be made available by the authors, without undue reservation.

## Author contributions

The author confirms being the sole contributor of this work and has approved it for publication.

## Conflict of interest

The author declares that the research was conducted in the absence of any commercial or financial relationships that could be construed as a potential conflict of interest.

## Publisher's note

All claims expressed in this article are solely those of the authors and do not necessarily represent those of their affiliated organizations, or those of the publisher, the editors and the reviewers. Any product that may be evaluated in this article, or claim that may be made by its manufacturer, is not guaranteed or endorsed by the publisher.

## Footnotes

^1^For reasons of completeness, it is worth mentioning that researchers can also use Bayesian SEM (BSEM; Muthén and Asparouhov, 2012) and set-ESEM (Marsh et al., 2020).

^2^Despite the widespread use of goodness-of-fit indices to evaluate model fit, many scholars warn against the use of fixed cutoff values (Marsh et al., 2004). McNeish and Wolf (2022) propose dynamic fit index cutoffs as an alternative.

## References

[B1] ^*^ Appiah R. Schutte L. Fadiji A. W. Wissing M. P. Cromhout A. (2020). Factorial validity of the Twi versions of five measures of mental health and well-being in Ghana. PLoS ONE 15, e0236707. 10.1371/journal.pone.0236707 32780773 PMC7418998

[B2] ^*^ Appleton P. R. Ntoumanis N. Quested E. Viladrich C. Duda J. L. (2016). Initial validation of the coach-created empowering and disempowering motivational climate questionnaire (EDMCQ-C). Psychol. Sport Exerc. 22, 53–65. 10.1016/j.psychsport.2015.05.008

[B3] ^*^ Arens A. K. Jansen M. Preckel F. Schmidt I. Brunner M. (2021). The structure of academic self-concept: a methodological review and empirical illustration of central models. Rev. Educ. Res. 91, 34–72. 10.3102/0034654320972186

[B4] ^*^ Arens A. K. Morin A. J. S. (2017). Improved representation of the self-perception profile for children through bifactor exploratory structural equation modeling. Am. Educ. Res. J. 54, 59–87. 10.3102/0002831216666490

[B5] ^*^ Arias V. B. Jenaro C. Ponce F. P. (2018). Testing the generality of the general factor of personality: an exploratory bifactor approach. Pers. Individ. Dif. 129, 17–23. 10.1016/j.paid.2018.02.042

[B6] ^*^ Arias V. B. Ponce F. P. Martinez-Molina A. Arias B. Nunez D. (2016). General and specific attention-deficit/hyperactivity disorder factors of children 4 to 6 years of age: an exploratory structural equation modeling approach to assessing symptom multidimensionality. J. Abnorm. Psychol. 125, 125–137. 10.1037/abn0000115 26726819

[B7] Asparouhov T. Muthén B. (2009). Exploratory structural equation modeling. Struct. Equ. Model. 16, 397–438. 10.1080/10705510903008204

[B8] Bandalos D. L. Finney S. J. (2018). “Exploratory and confirmatory factor analysis,” in Quantitative Methods in the Social and Behavioral Sciences: A Guide for Researchers and Reviewers, 2nd Edn, eds G. R. Hancock, and R. O. Mueller (New York, NY: Routledge), 93–114.

[B9] ^*^ Barbaranelli C. Fida R. Paciello M. Tramontano C. (2018). ‘Possunt, quia posse videntur': THEY can because they think they can. Development and validation of the work self-efficacy scale: evidence from two studies. J. Voc. Behav. 106, 249–269. 10.1016/j.jvb.2018.01.006

[B10] ^*^ Bédard-Thom C. Guay F. (2018). Mental toughness among high school students: a test of its multidimensionality and nomological validity with academic achievement and preference for difficult tasks. Soc. Psychol. Educ. 21, 827–848. 10.1007/s11218-018-9437-y

[B11] ^*^ Benda J. (2018). Alternative Models of the Czech Version of the Self-Compassion Scale (SCS-26-CZ) (Prague: University of Prague). Unpublished manuscript.

[B12] Bentler P. M. (1990). Comparative fit indexes in structural models. Psychol. Bull. 107, 238–246. 10.1037/0033-2909.107.2.238 2320703

[B13] Bentler P. M. (1995). EQS Structural Equations Program Manual. Encino, CA: Multivariate Software Inc.

[B14] Bentler P. M. Bonett D. G. (1990). Significance tests and goodness of fit in the analysis of covariance structures. Psychol. Bull. 88, 588–606. 10.1037/0033-2909.88.3.588

[B15] ^*^ Bhavsar N. Bartholomew K. J. Quested E. Gucciardi D. F. Thøgersen-Ntoumani C. Reeve J. . (2020). Measuring psychological need states in sport: theoretical considerations and a new measure. Psychol. Sport Exerc. 47, 101617. 10.1016/j.psychsport.2019.101617

[B16] ^*^ Bhavsar N. Ntoumanis N. Quested E. Gucciardi D. F. Thøgersen-Ntoumani C. Ryan R. M. . (2019). Conceptualizing and testing a new tripartite measure of coach interpersonal behaviors. Psychol. Sport Exerc. 44, 107–120. 10.1016/j.psychsport.2019.05.006

[B17] ^*^ Bianchi R. (2020). Do burnout and depressive symptoms form a single syndrome? Confirmatory factor analysis and exploratory structural equation modeling bifactor analysis. J. Psychosom. Res. 131, 109954. 10.1016/j.jpsychores.2020.109954 32036062

[B18] ^*^ Bianchi R. Schonfeld I. S. (2020). The occupational depression inventory: a new tool for clinicians and epidemiologists. J. Psychosom. Res. 138, 110249. 10.1016/j.jpsychores.2020.110249 32977198

[B19] ^*^ Bianchi R. Verkuilen J. (2021). “Green et al. paranoid thoughts scale”: French validation and development of a brief version. Pers. Indiv. Diff. 171, 110554. 10.1016/j.paid.2020.110554

[B20] ^*^ Borges L. Nunes Baptista M. de Oliveira Serpa A. L. (2017). Structural analysis of depression indicators scale-children and adolescents (BAID-IJ): a bifactor-ESEM approach. Trends Psychol. 25, 545–552. 10.9788/TP2017.2-08

[B21] ^*^ Burgueño R. González-Cutre D. Sevil-Serrano J. Herrador-Colmenero M. SeguraDíaz J. M. Medina-Casaubón J. . (2020a). Validation of the basic psychological need satisfaction in active commuting to and from school (BPNS-ACS) scale in Spanish young people. J. Transp. Health 16, 100825. 10.1016/j.jth.2020.100825

[B22] ^*^ Burgueño R. Macarro-Moreno J. Medina-Casaubón J. (2020b). Psychometry of the multidimensional perceived autonomy support scale in physical education with Spanish secondary school students. SAGE Open 10, 1–12. 10.1177/2158244019901253

[B23] ^*^ Burk C. L. Wiese B. S. (2018). Professor or manager? A model of motivational orientations applied to preferred career paths. J. Res. Pers. 75, 113–132. 10.1016/j.jrp.2018.06.002

[B24] ^*^ Calkins C. M. (2018). Developing and measuring faculty motivation to teach in higher education. (Doctoral thesis), University of Nevada, Reno (Nevada).

[B25] ^*^ Cano-García F. J. Muñoz-Navarro R. Abad A. S. Moretti L. S. Medrano L. A. Ruiz-Rodríguez P. . (2020). Latent structure and factor invariance of somatic symptoms in the patient health questionnaire (PHQ−15). J. Affect. Disord. 261, 21–29. 10.1016/j.jad.2019.09.077 31600584

[B26] ^*^ Cece V. Lienhart N. Nicaise V. Guillet-Descas E. Martinent G. (2019). Longitudinal sport motivation among young athletes in intensive training settings: using methodological advances to explore temporal structure of youth behavioral regulation in sport questionnaire scores. J. Sport Exerc. Psychol. 41, 24–35. 10.1123/jsep.2017-0194 30909847

[B27] Cheung M. W.-L. Chan W. (2005). Meta-analytic structural equation modeling: a two-stage approach. Psychol. Methods 10, 40–64. 10.1037/1082-989X.10.1.40 15810868

[B28] ^*^ Choisay F. Fouquereau E. Coillot H. Chevalier S. (2021). Validation of the french psychological capital questionnaire (F–PCQ−24) and its measurement invariance using bifactor exploratory structural equation modeling framework. Mil. Psychol. 33, 50–65. 10.1080/08995605.2020.1852873 PMC10013531 38536364

[B29] ^*^ Chung C. M. Liao X. L. Song H. R. Lee T. H. (2016). Bifactor approach to modeling multidimensionality of physical self-perception profile. Meas. Phys. Educ. Exerc. Sci. 20, 1–15. 10.1080/1091367X.2015.1081594

[B30] ^*^ Clifton J. Seehuus M. Parent J. Pichler E. Fondacaro K. (2020). Emotional responding: integration of multiple constructs and association with psychological health. J. Clin. Psychol. 76, 699–715. 10.1002/jclp.22885 31714614

[B31] ^*^ Cromhout A. (2020). Measuring and understanding eudaimonic well-being: A bifactor exploratory structural equation modelling approach. (Doctoral thesis), North-West University, Boloka. Available online at: http://hdl.handle.net/10394/36666 (accessed June 29, 2021).

[B32] ^*^ Cronin L. D. Allen J. (2017). Development and initial validation of the life skills scale for sport. Psychol. Sport Exerc. 28, 105–119. 10.1016/j.psychsport.2016.11.001

[B33] Dai S. S. Xu H. G. Chen F. F. (2019). A hierarchical measurement model of perceived resilience of urban tourism destination. Soc. Indic. Res. 145, 777–804. 10.1007/s11205-019-02117-9

[B34] ^*^ Decroos S. Lines R. Morgan P. B. C. Fletcher D. Sarkar M. Fransen K. . (2017). Development and validation of the characteristics of resilience in sports teams inventory. Sport Exerc. Perform. Psychol. 6, 158–178. 10.1037/spy0000089

[B35] ^*^ Deemer E. D. Smith J. L. Thoman D. B. Chase J. P. (2014). Precision in career motivation assessment: testing the subjective science attitude change measures. J. Car. Assess. 22, 489–504. 10.1177/1069072713498683

[B36] ^*^ Deller J. Perrotte J. Wainwright K. Brunsman J. Osman A. (2020). Dimensionality, reliability, invariance, and validity of the multidimensional social anxiety response inventory−21 (MSARI−21). J. Pers. Assess. 102, 527–537. 10.1080/00223891.2019.1569529 30907638 PMC6761042

[B37] ^*^ Díaz-Morales J. F. Parra-Robledo Z. (2018). Age and sex differences in morningness/eveningness along the life span: a cross-sectional study in Spain. J. Genet. Psychol. 179, 71–84. 10.1080/00221325.2018.1424706 29424669

[B38] ^*^ Dierendonck C. Tóth-Király I. Morin A. J. S. Kerger S. Milmeister P Poncelet D. (2022). Testing associations between global and specific levels of student academic motivation and engagement in the classroom. J. Exp. Educ. 10.1080/00220973.2021.1913979

[B39] ^*^ Doherty A. S. Mallett J. Leiter M. P. McFadden P. (2021). Measuring burnout in social work factorial validity of the Maslach burnout inventory – human Services Survey. Euro. J. Psychol. Assess. 37, 6–14. 10.1027/1015-5759/a000568

[B40] ^*^ Dominguez-Lara S. Fernández-Arata M. Cesar M. S. Navarro-Loli J. S. Calderón-De la Cruz G. (2019). Escala de autoeficacia docente: análisis estructural e invarianza de medición en docentes peruanos de escuelas públicas [Teacher's self-efficacy scale: Structural analysis and measurement invariance in Peruvian teachers of public schools]. Rev. Argent. Cienc. Comport. 11, 61–72. 10.32348/1852.4206.v11.n3.24624

[B41] Eid M. Geiser C. Koch T. Heene M. (2017). Anomalous results in G-factor models: explanations and alternatives. Psychol. Methods 22, 541–562. 10.1037/met0000083 27732052

[B42] ^*^ Esnaola I. Arias V. B. Freeman J. Wang Y. Arias B. (2018). Validity evidence based on internal structure of scores of the emotional quotient inventory: youth version short (EQ-i: YV-S) in a Chinese sample. J. Psychoeduc. Assess. 36, 576–587. 10.1177/0734282916689439

[B43] ^*^ Espinoza J. A. Meyer J. P. Anderson B. K. Vaters C. Politis C. (2018). Evidence for a bifactor structure of the scales of psychological well-being using exploratory structural equation modeling. J. Well Being Assess. 2, 21–40. 10.1007/s41543-018-0008-y

[B44] ^*^ Fadda D. Quevedo-Aguado M. P. Cuesta M. H. B. Scalas L. F. (2020a). The multidimensional and hierarchical nature of the questionnaire for eudaimonic wellbeing: a bifactor–ESEM representation in a Spanish Sample. Front. Psychol. 11, 422. 10.3389/fpsyg.2020.00422 32218760 PMC7078344

[B45] ^*^ Fadda D. Scalas L. F. Meleddu M. Morin A. J. S. (2017). A bifactor–ESEM representation of the questionnaire for eudaimonic wellbeing. Pers. Individ. Dif. 116, 216–222. 10.1016/j.paid.2017.04.062

[B46] ^*^ Fadda D. Scalas L. F. Morin A. J. S. Marsh H. W. Gaspard H. (2020b). Value beliefs about math: a bifactor–ESEM representation. Euro. J. Psychol. Assess. 36, 259–268. 10.1027/1015-5759/a000513

[B47] ^*^ Ferentinos P. Yotsidi V. Porichi E. Douzenis A. Papageorgiou C. Stalikas A. (2019). Well-being in patients with affective disorders compared to nonclinical participants: a multi-model evaluation of the mental health continuum–short form. J. Clin. Psychol. 75, 1585–1612. 10.1002/jclp.22780 30995352

[B48] ^*^ Fernandez-Rio J. Cecchini J. A. Morgan K. Méndez-Giménez A. Lloyd R. (2022). Validation of the cooperative learning scale and cooperation global factor using bifactor structural equation modelling. Psicol. Educ. 28, 91–97. 10.5093/psed2021a2

[B49] ^*^ Fresno A. Arias V. Nunez D. Spencer R. Ramos N. Espinoza C. . (2020). Using exploratory structural equation modeling (ESEM) to examine the internal structure of posttraumatic stress disorder symptoms. Span. J. Psychol. 23, e48. 10.1017/SJP.2020.46 33176894

[B50] ^*^ Frutos J. (2019). Evaluación multidimensional de los factores generales y específicos del TDAH en población infantial mediante el enfoque bifactor-ESEM [multidimensional evaluation of the general and specific facors of ADHD in children population with the bifactor-ESEM approach]. Rev. Argent. Clín. Psicol. 28, 967–980. 10.51668/bp.8321105s

[B51] ^*^ Frutos J. (2021). Análisis multidimensional del trastorno por déficit de atención e hiperactividad (TDAH) mediante el método bifactor-ESEM [multidimensional analysis of attention deficit hyperactivity disorder (ADHD) using the bifactor–ESEM method]. Behav. Psychol. 29, 95–110.

[B52] ^*^ García-Garzón E. Nieto M. D. Garrido L. E. Abad F. J. (2020). Bi-factor exploratory structural equation modeling done right: Using the SLiDapp application. Psicothema 32, 607–614. 10.7334/psicothema2020.179 33073768

[B53] ^*^ Garn A. C. (2017). Multidimensional measurement of situational interest in physical education: application of bifactor exploratory structural equation modeling. J. Teach. Phys. Educ. 36, 323–339. 10.1123/jtpe.2017-0035

[B54] ^*^ Garn A. C. Morin A. J. S. Lonsdale C. (2019). Basic psychological need satisfaction toward learning: a longitudinal test of mediation using bifactor exploratory structural equation modeling. J. Educ. Psychol. 111, 354–372. 10.1037/edu0000283

[B55] ^*^ Garn A. C. Webster E. K. (2021). Bifactor structure and model reliability of the test of gross motor development−3rd edition. J. Sci. Med. Sport 24, 67–73. 10.1016/j.jsams.2020.08.009 32919885

[B56] ^*^ Gegenfurtner A. Quesada-Pallarès C. (2022). Toward a multidimensional conceptualization of motivation to transfer training: validation of the transfer motivation questionnaire from a self-determination theory perspective using bifactor-ESEM. Stud. Educ. Eval. 73, 101116. 10.1016/j.stueduc.2021.101116

[B57] ^*^ Gillet N. Caesens G. Morin A. J. S. Stinglhamber F. (2019). Complementary variable– and person-centred approaches to the dimensionality of work engagement: a longitudinal investigation. Euro. J. Work Organ. Psychol. 28, 239–258. 10.1080/1359432X.2019.1575364

[B58] ^*^ Gillet N. Morin A. J. S. Huart I. Colombat P. Fouquereau E. (2020). The forest and the trees: investigating the globality and specificity of employees' basic need satisfaction at work. J. Pers. Assess. 102, 702–713. 10.1080/00223891.2019.1591426 31012751

[B59] ^*^ Giotsa A. Kyriazos T. A. (2019). Early childhood acceptance rejection questionnaire: psychometric properties of the Greek version. Psychology 10, 722–741. 10.4236/psych.2019.105047

[B60] ^*^ Gomez R. Stavropoulos V. (2021). Confirmatory factor analysis and exploratory structural equation modeling of the structure of attention-deficit/hyperactivity disorder symptoms in adults. Assessment 28, 1570–1582. 10.1177/1073191120905892 32062977

[B61] ^*^ Gomez R. Stavropoulos V. Griffiths M. D. (2020a). Confirmatory factor analysis and exploratory structural equation modelling of the factor structure of the depression anxiety and stress scales−21. PLoS ONE 15, e0233998. 10.1371/journal.pone.0233998 32502165 PMC7274426

[B62] ^*^ Gomez R. Stavropoulos V. Zarate D. Palikara O. (2021). Symptom checklist-90–revised: a structural examination in relation to family functioning. PLoS ONE 16, e0247902. 10.1371/journal.pone.0247902 33711019 PMC7954339

[B63] ^*^ Gomez R. Watson S. Stavropoulos V. (2020b). Personality inventory for DSM−5, brief form: factor structure, reliability, and coefficient of congruence. Pers. Disord. Theory Res. Treat. 11, 69–77. 10.1037/per0000364 31670543

[B64] ^*^ Goodboy A. K. Bolkan S. Brisini K. Solomon D. H. (2021). Relational uncertainty within relational turbulence theory: the bifactor exploratory structural equation model. J. Commun. 71, 403–430.. 10.1093/joc/jqab009

[B65] ^*^ Gordeeva T. O. Sychev O. A. Lynch M. F. (2020). The construct validity of the Russian version of the modified academic self-regulation questionnaire (SRQ–A) among elementary and middle school children. Psychol. Russia 13, 16–34. 10.11621/pir.2020.0308

[B66] ^*^ Grygiel P. Humenny G. Rebisz S. (2019). Using the De Jong Gierveld loneliness scale with early adolescents: factor structure, reliability, stability, and external validity. Assessment 26, 151–165. 10.1177/1073191116682298 27932403

[B67] ^*^ Gu H. L. Wen Z. L. Fan X. T. (2017). Structural validity of the Machiavellian personality scale: a bifactor exploratory structural equation modeling approach. Pers. Individ. Dif. 105, 116–123. 10.1016/j.paid.2016.09.042

[B68] ^*^ Gu H. L. Wen Z. L. Fan X. T. (2020). Investigating the multidimensionality of the work-related flow inventory (WOLF): a bifactor exploratory structural equation modeling framework. Front. Psychol. 11, 740. 10.3389/fpsyg.2020.00740 32435216 PMC7218516

[B69] ^*^ Guay F. Bureau J. S. (2018). Motivation at school: differentiation between and within school subjects matters in the prediction of academic achievement. Contemp. Educ. Psychol. 54, 42–54. 10.1016/j.cedpsych.2018.05.004

[B70] ^*^ Guay F. Morin A. J. S. Litalien D. Howard J. L. Gilbert W. (2021). Trajectories of self-determined motivation during the secondary school: a growth mixture analysis. J. Educ. Psychol. 113, 390–410. 10.1037/edu0000482

[B71] ^*^ Gucciardi D. F. Weixian J. C. Gibson W. Ntoumanis N. Ng L. (2020). Motivational climate in the classroom factorial and convergent validity: evidence of the need-supportive behaviors scale with health science students. Euro. J. Psychol. Assess. 36, 324–335. 10.1027/1015-5759/a000524

[B72] ^*^ Halamová J. Kanovský M. Pacúchová M. (2020). Psychometric analysis of the Slovak version of the compassionate engagement and action scales. J. Psychol. Educ. Res. 28, 64–80.

[B73] ^*^ Hanley A. W. Bernstein A. Nakamura Y. Hadash Y. Rojas J. Tennant K. E. . (2020). The metacognitive processes of decentering scale: development and initial validation of trait and state versions. Psychol. Assess. 32, 956–971. 10.1037/pas0000931 32700920 PMC8647764

[B74] ^*^ Haugen T. Peters D. M. Ommundsen Y. Martin L. J. Stenling A. Høigaard R. (2021). Psychometric evaluation of the Norwegian versions of the modified group environment questionnaire and the youth sport environment questionnaire. Meas. Phys. Educ. Exerc. Sci. 25, 365–378. 10.1080/1091367X.2021.1917414

[B75] ^*^ Hoi V. N. Hang H. L. (2021). The structure of student engagement in online learning: a bi-factor exploratory structural equation modelling approach. J. Comput. Assist. Learn. 37,1141–1153. 10.1111/jcal.12551

[B76] ^*^ Høstmælingen A. Ulvenes P. Nissen-Lie H. A. Eielsen M. Wampold B. E. (2021). Do self-criticism and somatic symptoms play a key role in chronic depression? Exploring the factor structure of beck depression inventory–II in a sample of chronically depressed inpatients. J. Affect. Disord. 283, 317–324. 10.1016/j.jad.2021.01.066 33578344

[B77] ^*^ Howard J. L. Gagn,é M. Van den Broeck A. Guay F. Chatzisarantis N. Ntoumanis N. . (2020). A review and empirical comparison of motivation scoring methods: an application to self-determination theory. Motiv. Emot. 44, 534–548. 10.1007/s11031-020-09831-9

[B78] ^*^ Howard J. L. Gagné M. Morin A. J. S. Forest J. (2018). Using bifactor exploratory structural equation modeling to test for a continuum structure of motivation. J. Manage. 44, 2638–2664. 10.1177/0149206316645653

[B79] ^*^ Howard J. L. Morin A. J. S. Gagné M. (2021). A longitudinal analysis of motivation profiles at work. Motiv. Emot. 45, 39–59. 10.1007/s11031-020-09852-4

[B80] Hu L. T. Bentler P. M. (1999). Cutoff criteria for fit indexes in covariance structure analysis: conventional criteria versus new alternatives. Struct. Equ. Model. 6, 1–55. 10.1080/10705519909540118

[B81] ^*^ Hukkelberg S. Ogden T. (2019). General and specific factors of working alliance in parent training: a bifactor exploratory structural equation modelling approach. Psychother. Res. 29, 267–276. 10.1080/10503307.2017.1330574 28610475

[B82] ^*^ Hukkelberg S. Ogden T. (2020). What is social competence? An investigation into the concept among children with antisocial behaviours. Emot. Behav. Diff. 25, 80–93. 10.1080/13632752.2019.1687168

[B83] ^*^ Hukkelberg S. S. (2019). A reexamination of child problem behaviors as measured by ECBI: factor structure and measurement invariance across two parent training interventions. Assessment 26, 1270–1281. 10.1177/1073191117706022 28470086

[B84] ^*^ Huyghebaert-Zouaghi T. Berjot S. Cougot B. Gillet N. (2021a). Psychological and relational conditions for job crafting to occur. Stress Health 37, 516–527. 10.1002/smi.3014 33314676

[B85] ^*^ Huyghebaert-Zouaghi T. Caesens G. Sandrin É. Gillet N. (2022). Workaholism and work engagement: an examination of their psychometric multidimensionality and relations with employees' functioning. Curr. Psychol. 10.1007/s12144-021-01820-6

[B86] ^*^ Huyghebaert-Zouaghi T. Ntoumanis N. Berjot S. Gillet N. (2021b). Advancing the conceptualization and measurement of psychological need states: a 3 x 3 model based on self-determination theory. J. Car. Assess. 29, 396–421. 10.1177/1069072720978792

[B87] ^*^ Isoard-Gautheur S. Martinent G. Guillet-Descas E. Trouilloud D. Cece V. Mette A. (2018). Development and evaluation of the psychometric properties of a new measure of athlete burnout: the athlete burnout scale. Int. J. Stress Manag. 25, 108–123. 10.1037/str0000083

[B88] ^*^ Jaotombo F. F. (2019). Le fonctionnement optimal psychologique: apports conceptuels et méthodologiques [Optimal psychological functioning: conceptual and methodological contributions]. Psychol. Trav. Organ. 25, 281–300. 10.1016/j.pto.2019.06.001

[B89] ^*^ Jastrzebski J. Rogoza R. Slaski S. (2020). The hierarchical structure of fear of personal death: from the general factor to specific forms. Psicol. Reflexao Crit. 33, 16. 10.1186/s41155-020-00152-x PMC7371767 32691175

[B90] Jöreskog K. G. (1969). A general approach to confirmatory maximum likelihood factor analysis. Psychometrika 34, 183–202. 10.1007/BF02289343

[B91] ^*^ Jovanovic V. Gavrilov-Jerkovic V. Lazic M. (2021). Can adolescents differentiate between depression, anxiety and stress? Testing competing models of the depression anxiety stress scales (DASS−21). Curr. Psychol. 40, 6045–6056. 10.1007/s12144-019-00540-2

[B92] ^*^ Junga Y. M. Witthoft M. Weck F. (2019). Assessing therapist development: reliability and validity of the supervisee levels questionnaire (SLQ–R). J. Clin. Psychol. 75, 1658–1672. 10.1002/jclp.22794 31009551

[B93] ^*^ Kartal S. K. (2020). Examining factors for the academic motivation based on the confirmatory, the exploratory, and the bifactor exploratory structural equation modeling. Int. J. Prog. Educ. 16, 192–204. 10.29329/ijpe.2020.228.14

[B94] ^*^ Kawabata M. Pavey T. G. Coulter T. J. (2021). Evolving the validity of a mental toughness measure: refined versions of the mental toughness questionnaire-48. Stress Health 37, 378–391. 10.1002/smi.3004 33145967

[B95] Koo T. K. Li M. Y. (2016). A guideline for selecting and reporting intraclass correlation coefficients for reliability research. J. Chiropr. Med. 15, 155–163. 10.1016/j.jcm.2016.02.012 27330520 PMC4913118

[B96] ^*^ Korol L. Fietzer A. W. Ponterotto J. G. (2018). The relationship between multicultural personality, intergroup contact, and positive outgroup attitudes toward Asian Americans. Asian Am. J. Psychol. 9, 200–210. 10.1037/aap0000107

[B97] ^*^ Krafft A. Guse T. Maree D. (2020). Distinguishing perceived hope and dispositional optimism: theoretical foundations and empirical findings beyond future expectancies and cognition. J. Well Being Assess. 4, 217–243. 10.1007/s41543-020-00030-4

[B98] ^*^ Kyriazos T. A. Stalikas A. Prassa K. Galanakis M. Flora K. Chatzilia V. (2018b). The flow short scale (FSS) dimensionality and what MIMIC shows on heterogeneity and invariance. Psychology 9, 1357–1382. 10.4236/psych.2018.96083

[B99] ^*^ Kyriazos T. A. Stalikas A. Prassa K. Yotsidi V. (2018a). A 3-faced construct validation and a bifactor subjective well-being model using the scale of positive and negative experience, Greek version. Psychology 9, 1143–1175. 10.4236/psych.2018.95071

[B100] ^*^ Lahey B. B. Zald D. H. Perkins S. F. Villalta-Gil V. Werts K. B. Van Hulle C. A. . (2018). Measuring the hierarchical general factor model of psychopathology in young adults. Int. J. Methods Psychiatr. Res. 27, e1593. 10.1002/mpr.1593 PMC5834349 28990308

[B101] ^*^ Lamborn P. Cramer K. M. Riberdy A. (2018). The structural validity and measurement invariance of the mental health continuum–short form (MHC–SF) in a large Canadian same. J. Well Being Assess 2, 1–19. 10.1007/s41543-018-0007-z

[B102] Landis J. R. Koch G. G. (1977). The measurement of observer agreement for categorical data. Biometrics 33, 159–174. 10.2307/2529310 843571

[B103] ^*^ Lauriola M. Donati M. A. Trentini C. Tomai M. Pontone S. Baker R. (2021). The structure of the Emotional processing scale (EPS-25): an exploratory structural equation modeling analysis using medical and community samples. Euro. J. Psychol. Assess. 37, 423–432. 10.1027/1015-5759/a000632

[B104] ^*^ Lecerf T. Canivez G. L. (2018). Complementary exploratory and confirmatory factor analyses of the French WISC–V: analyses based on the standardization sample. Psychol. Assess. 30, 793–808. 10.1037/pas0000526 29283593

[B105] ^*^ Lee P. Mahoney K. T. Lee S. (2017). An application of the exploratory structural equation modeling framework to the study of personality faking. Pers. Individ. Dif. 119, 220–226. 10.1016/j.paid.2017.07.029

[B106] ^*^ Leo F. M. González-Ponce I. Sánchez-Oliva D. Pulido J. J. García-Calvo T. (2017). Role ambiguity: translation to Spanish and analysis of scale structure. Small Group Res. 48, 365–385. 10.1177/1046496417706554

[B107] Leys C. Ley C. Klein O. Bernard P. Licata L. (2013). Detecting outliers: do not use standard deviation around the mean, use absolute deviation around the median. J. Exp. Soc. Psychol. 49, 764–766. 10.1016/j.jesp.2013.03.013

[B108] ^*^ Litalien D. Morin A. J. S. Gagn,é M. Vallerand R. J. Losier G. F. Ryan R. M. (2017). Evidence of a continuum structure of academic self–determination: a two-study test using a bifactor-ESEM representation of academic motivation. Contemp. Educ. Psychol. 51, 67–82. 10.1016/j.cedpsych.2017.06.010

[B109] ^*^ Lohbeck A. Petermann F. (2019). Factorial validity of the anxiety questionnaire for students (AFS): bifactor modeling and measurement invariance. J. Psychoeduc. Assess. 37, 770–781. 10.1177/0734282918794834

[B110] ^*^ Longo Y. Jovanovic V. de Carvalho J. S. Karas D. (2020). The general factor of well-being: multinational evidence using bifactor ESEM on the mental health continuum–short form. Assessment 27, 596–606. 10.1177/1073191117748394 29281897

[B111] ^*^ Maïano C. Morin A. J. S. Aimé A. Lepage G. Bouchard S. (2021). Psychometric properties of the body checking questionnaire (BCQ) and of the body checking cognitions scale (BCCS): a bifactor-exploratory structural equation modeling approach. Assessment 28, 632–646. 10.1177/1073191119858411 31328530

[B112] Marsh H. W. (2007). “Application of confirmatory factor analysis and structural equation modeling in sport/exercise psychology,” in Handbook of Sport Psychology, eds G. Tenenbaum, and R. C. Eklund (New York, NY: Wiley), 774–798. 10.1002/9781118270011.ch35

[B113] Marsh H. W. Guo J. Dicke T. Parker P. D. Craven R. G. (2020). Confirmatory factor analysis (CFA), exploratory structural equation modeling (ESEM), and set-ESEM: optimal balance between goodness of fit and parsimony. Multivariate Behav. Res. 55, 102–119. 10.1080/00273171.2019.1602503 31204844

[B114] Marsh H. W. Hau K.-T. Wen Z. (2004). In search of golden rules: comment on hypothesis-testing approaches to setting cutoff values for fit indexes and dangers in overgeneralizing Hu and Bentler's (1999) findings. Struct. Equ. Model. 11, 320–341. 10.1207/s15328007sem1103_2

[B115] Marsh H. W. Morin A. J. S. Parker P. D. Kaur G. (2014). Exploratory structural equation modeling: an integration of the best features of exploratory and confirmatory factor analysis. Annu. Rev. Clin. Psychol. 10, 85–110. 10.1146/annurev-clinpsy-032813-153700 24313568

[B116] Marsh H. W. Muthén B. Asparouhov A. Lüdtke O. Robitzsch A. Morin A. J. S. . (2009). Exploratory structural equation modeling, integrating CFA and EFA: application to students' evaluations of university teaching. Struct. Equ. Model. 16, 439–476. 10.1080/10705510903008220

[B117] ^*^ McKay M. T. Perry J. L. Percy A. Cole J. C. (2016). Evidence for the reliability and validity, and some support for the practical utility of the two-factor consideration of future consequences scale−14. Pers. Individ. Dif. 98, 133–136. 10.1016/j.paid.2016.03.097

[B118] ^*^ McLarnon M. J. W. Tarraf R. C. (2017). The dark triad: specific or general sources of variance? A bifactor exploratory structural equation modeling approach. Pers. Indiv. Diff. 112, 67–73. 10.1016/j.paid.2017.02.049

[B119] ^*^ McLarnon M. J. W. Tarraf R. C. (2021). Getting to the core: how “(dis)honest” is the core of the dark triad? Pers. Individ. Dif. 171, 110545. 10.1016/j.paid.2020.110545

[B120] McNeish D. Wolf M. G. (2022). Dynamic fit index cutoffs for confirmatory factor analysis models. Psychol. Methods. 10.1037/met0000425 34694832

[B121] ^*^ Méndez-Giménez A. Cecchini-Estrada J. A. Rodríguez-González P. (2020). Competencia percibida (tridimensional), regulaciones motivacionales y autoeficacia en educación física [perceived competence (three-dimensional), motivational regulations and self-efficacy in physical education]. Rev. Latinoam. Psicol. 52, 51–62. 10.14349/rlp.2020.v52.6

[B122] Miller J. (1991). Reaction time analysis with outlier exclusion: bias varies with sample size. Q. J. Exp. Psychol. 43, 907–912. 10.1080/14640749108400962 1775668

[B123] ^*^ Milton D. Appleton P. R. Bryant A. Duda J. L. (2018). Initial validation of the teacher-created empowering and disempowering motivational climate questionnaire in physical education. J. Teach. Phys. Educ. 37, 340–351. 10.1123/jtpe.2018-0119

[B124] ^*^ Morin A. J. S. Arens A. K. Marsh H. W. (2016a). A bifactor exploratory structural equation modeling framework for the identification of distinct sources of construct-relevant psychometric multidimensionality. Struct. Equ. Model. 23, 116–139. 10.1080/10705511.2014.961800

[B125] ^*^ Morin A. J. S. Arens A. K. Tran A. Caci H. (2016b). Exploring sources of construct-relevant multidimensionality in psychiatric measurement: a tutorial and illustration using the composite scale of morningness. Int. J. Methods Psychiatr. Res. 25, 277–288. 10.1002/mpr.1485 26265387 PMC6860252

[B126] ^*^ Morin A. J. S. Boudrias J. S. Marsh H. W. Madore I. Desrumaux P. (2016c). Further reflections on disentangling shape and level effects in person-centered analyses: an illustration exploring the dimensionality of psychological health. Struct. Equ. Model. 23, 438–454. 10.1080/10705511.2015.1116077

[B127] ^*^ Morin A. J. S. Boudrias J. S. Marsh H. W. McInerney D. M. Dagenais–Desmarais V. Madore I. . (2017). Complementary variable- and person-centered approaches to the dimensionality of psychometric constructs: application to psychological wellbeing at work. J. Bus. Psychol. 32, 395–419. 10.1007/s10869-016-9448-7

[B128] Morin A. J. S. Marsh H. W. Nagengast B. (2013). “Exploratory structural equation modeling,” in Structural Equation Modeling: A Second Course, 2nd Edn, eds G. R. Hancock, and R. O. Mueller (Charlotte, NC), 395–436.

[B129] Morin A. J. S. Myers N. D. Lee S. (2020). “Modern factor analytic techniques: bifactor models, exploratory structural equation modeling (ESEM) and bifactor-ESEM,” in Handbook of Sport Psychology, Vol. 2, 4th Edn, eds G. Tenenbaum, and R. C. Eklund (London: Wiley), 1044–1073. 10.1002/9781119568124.ch51

[B130] Muthén B. Asparouhov T. (2012). Bayesian structural equation modeling: a more flexible representation of substantive theory. Psychol. Methods 17, 313–335. 10.1037/a0026802 22962886

[B131] ^*^ Myers N. D. Park S. E. Lefevor G. T. Dietz S. Prilleltensky I. Prado G. J. (2016). Measuring multidimensional subjective well-being with the I COPPE scale in a hispanic sample. Meas. Phys. Educ. Exerc. Sci. 20, 230–243. 10.1080/1091367X.2016.1226836 33828400 PMC8023353

[B132] ^*^ Neff K. D. Bluth K. Tóth-Király I. Davidson O. Knox M. C. Williamson Z. . (2021a). Development and validation of the self-compassion scale for youth. J. Pers. Assess. 103, 92–105. 10.1080/00223891.2020.1729774 32125190

[B133] ^*^ Neff K. D. Tóth-Király I. Colosimo K. (2018). Self-compassion is best measured as a global construct and is overlapping with but distinct from neuroticism: a response to Pfattheicher, Geiger, Hartung, Weiss, and Schindler (2017). Eur. J. Pers. 32, 371–392. 10.1002/per.2148

[B134] ^*^ Neff K. D. Tóth-Király I. Knox M. C. Kuchar A. Davidson O. (2021b). The development and validation of the state self–compassion scale (long- and short form). Mindfulness 12, 121–140. 10.1007/s12671-020-01505-4

[B135] ^*^ Neff K. D. Tóth-Király I. Yarnell L. M. Arimitsu K. Castilho P. Ghorbani N. . (2019). Examining the factor structure of the self-compassion scale in 20 diverse samples: support for use of a total score and six subscale scores. Psychol. Assess. 31, 27–45. 10.1037/pas0000629 30124303

[B136] ^*^ Ng V. Cao M. Y. Marsh H. W. Tay L. Seligman M. E. P. (2017). The factor structure of the values in action inventory of strengths (VIA–IS): an item–level exploratory structural equation modeling (ESEM) bifactor analysis. Psychol. Assess. 29, 1053–1058. 10.1037/pas0000396 27736126

[B137] ^*^ Nixon N. Guo B. L. Garland A. Kaylor-Hughes C. Nixon E. Morriss R. (2020). The bi-factor structure of the 17-item hamilton depression rating scale in persistent major depression; dimensional measurement of outcome. PLoS ONE 15, e0241370. 10.1371/journal.pone.0241370 33104761 PMC7588071

[B138] Page M. J. McKenzie J. E. Bossuyt P. M. Boutron I. Hoffmann T. C. Mulrow C. D. . (2021). The PRISMA 2020 statement: an updated guideline for reporting systematic reviews. BMJ 372, 71. 10.1136/bmj.n71 PMC8005924 33782057

[B139] ^*^ Part R. Perera H. N. Marchand G. C. Bernacki M. L. (2020). Revisiting the dimensionality of subjective task value: towards clarification of competing perspectives. Contemp. Educ. Psychol. 62, 101875. 10.1016/j.cedpsych.2020.101875

[B140] Pearson K. (1900). On the criterion that a given system of deviations from the probable in the case of a correlated system of variables is such that it can be reasonably supposed to have arisen from random sampling. Lond. Edinburgh Dublin Philos. Mag. J. Sci. 50, 157–175. 10.1080/14786440009463897

[B141] ^*^ Perera H. N. (2016). Construct validity of the social provisions scale: a bifactor exploratory structural equation modeling approach. Assessment 23, 720–733. 10.1177/1073191115589344 26063712

[B142] ^*^ Perera H. N. Ganguly R. (2018). Construct validity of scores from the Connor–Davidson resilience scale in a sample of postsecondary students with disabilities. Assessment 25, 193–205. 10.1177/1073191116646444 27141039

[B143] ^*^ Perera H. N. Izadikhah Z. O'Connor P. McIlveen P. (2018a). Resolving dimensionality problems with WHOQOL–BREF item responses. Assessment 25, 1014–1025. 10.1177/1073191116678925 27872348

[B144] ^*^ Perera H. N. Vosicka L. Granziera H. McIlveen P. (2018b). Towards an integrative perspective on the structure of teacher work engagement. J. Vocat. Behav. 108, 28–41. 10.1016/j.jvb.2018.05.006

[B145] ^*^ Perreira T. A. Morin A. J. S. Hebert M. Gillet N. Houle S. A. Berta W. (2018). The short form of the workplace affective commitment multidimensional questionnaire (WACMQ–S): a bifactor–ESEM approach among healthcare professionals. J. Vocat. Behav. 106, 62–83. 10.1016/j.jvb.2017.12.004

[B146] ^*^ Pirsoul T. Parmentier M. Nils F. (2022). One step beyond emotional intelligence measurement in the career development of adult learners: a bifactor exploratory structural equation modeling framework. Curr. Psychol. 10.1007/s12144-021-01772-x

[B147] ^*^ Pommier E. Neff K. D. Tóth-Király I. (2020). The development and validation of the compassion scale. Assessment 27, 21–39. 10.1177/1073191119874108 31516024

[B148] ^*^ Portoghese I. Porru F. Galletta M. Campagna M. Burdorf A. (2020). Stress among medical students: factor structure of the university stress scale among Italian students. BMJ Open 10, e035255. 10.1136/bmjopen-2019-035255 PMC7467511 32873666

[B149] ^*^ Ratelle C. F. Duchesne S. Guay F. Châteauvert G. B. (2018). Comparing the contribution of overall structure and its specific dimensions for competence–related constructs: a bifactor model. Contemp. Educ. Psychol. 54, 89–98. 10.1016/j.cedpsych.2018.05.005

[B150] ^*^ Reinhardt M. Horváth Z. Morgan A. Kökönyei G. (2020a). Well-being profiles in adolescence: psychometric properties and latent profile analysis of the mental health continuum model—a methodological study. Health Qual. Life Outcomes 18, 95. 10.1186/s12955-020-01332-0 32252785 PMC7137408

[B151] ^*^ Reinhardt M. Horváth Z. Tóth L. Kökönyei G. (2020b). A mentális egészség kontinuum skála rövid változatának hazai validációja. Magyar Pszichol. Szemle 75, 217–246. 10.1556/0016.2020.00014

[B152] Reinhold S. Gegenfurtner A. Lewalter D. (2018). Social support and motivation to transfer as predictors of training transfer: testing full and partial mediation using meta-analytic structural equation modeling. Int. J. Train. Dev. 22, 1–14. 10.1111/ijtd.12115

[B153] Reise S. P. (2012). The rediscovery of bifactor measurement models. Multivariate Behav. Res. 47, 667–669. 10.1080/00273171.2012.715555 24049214 PMC3773879

[B154] Rindskopf D. Rose T. (1988). Some theory and applications of confirmatory second-order factor analyses. Multivariate Behav. Res. 23, 51–67. 10.1207/s15327906mbr2301_3 26782257

[B155] ^*^ Rodenacker K. Hautmann C. Gortz–Dorten A. Dopfner M. (2017). The factor structure of ADHD—different models, analyses and informants in a bifactor framework. J. Psychopathol. Behav. Assess. 39, 92–102. 10.1007/s10862-016-9565-7

[B156] ^*^ Rodrigues F. Cid L. Teixeira D. Monteiro D. (2021). Re-applying the basic psychological needs in exercise scale to various Portuguese exercise groups: an analysis of bifactor models and contextual invariance. Percept. Mot. Skills 128, 1660–1683. 10.1177/00315125211016803 34000895

[B157] ^*^ Rodrigues F. Macedo R. Teixeira D. S. Cid L. Monteiro D. (2020). Motivation in sport and exercise: a comparison between the BRSQ and BREQ. Qual. Quant. 54, 1335–1350. 10.1007/s11135-020-00988-6

[B158] ^*^ Rogoza R. Thi K. H. T. Rózycka-Tran J. Piotrowski J. Zemojtel-Piotrowska M. (2018). Psychometric properties of the MHC–SF: an integration of the existing measurement approaches. J. Clin. Psychol. 74, 1742–1758. 10.1002/jclp.22626 29687455

[B159] ^*^ Sakakibara K. Shimazu A. Toyama H. Schaufeli W. B. (2020). Validation of the Japanese version of the burnout assessment tool. Front. Psychol. 11, 1819. 10.3389/fpsyg.2020.01819 32849072 PMC7431961

[B160] ^*^ Sánchez-Oliva D. Morin A. J. S. Teixeira P. J. Carraça E. V. Palmeira A. L. Silva M. N. (2017). A bifactor exploratory structural equation modeling representation of the structure of the basic psychological needs at work scale. J. Vocat. Behav. 98, 173–187. 10.1016/j.jvb.2016.12.001

[B161] ^*^ Scherer R. Nilsen T. Jansen M. (2016). Evaluating individual students' perceptions of instructional quality: an investigation of their factor structure, measurement invariance, and relations to educational outcomes. Front. Psychol. 7, 110. 10.3389/fpsyg.2016.00110 26903917 PMC4745267

[B162] ^*^ Schmid J. Steiner S. Rensch M. Middleton C. Seiler R. (2018). Psychometrische eigenschaften einer deutschsprachigen übersetzung des mental toughness inventory (MTI-D) [psychometric properties of a German translation of the mental toughness inventory (MIT-D)]. Diagnostica 64, 61–73. 10.1026/0012-1924/a000192

[B163] ^*^ Schutte L. Wissing M. P. (2017). Clarifying the factor structure of the mental health continuum short form in three languages: a bifactor exploratory structural equation modeling approach. Soc. Ment. Health 7, 142–158. 10.1177/2156869317707793

[B164] ^*^ Sellbom M. Tellegen A. (2019). Factor analysis in psychological assessment research: common pitfalls and recommendations. Psychol. Assess. 31, 1428–1441. 10.1037/pas0000623 31120298

[B165] ^*^ Sen R. Pleil A. M. Coon C. Shields A. L. (2015). Evaluating the dimensionality of complex pros using bifactor analysis within an exploratory structural equation modeling (ESEM) framework: An example using the patient-reported scar evaluation questionnaire (PR–SEQ). Quality of Life Research 24, 26.

[B166] Shi D. Lee T. Maydeu-Olivares A. (2019). Understanding the model size effect on SEM fit indices. Educ. Psychol. Meas. 79, 310–334. 10.1177/0013164418783530 30911195 PMC6425088

[B167] ^*^ Silverman A. L. Foregard M. Beard C. Björgvinsson T. (2018). Psychometric properties of the mental health continuum—short form in a psychiatric sample. Journal of Well Being Assess. 2, 57–73. 10.1007/s41543-018-0011-3

[B168] ^*^ Somma A. Borroni S. Drislane L. E. Patrick C. J. Fossati A. (2019). Modeling the structure of the triarchic psychopathy measure: comceptual, empirical, and analytical considerations. J. Pers. Disord. 33, 470–496. 10.1521/pedi_2018_32_354 30036170

[B169] Stanley T. D. Doucouliagos H. (2017). Neither fixed nor random: weighted least squares meta-regression. Res. Synth. Methods 8, 19–42. 10.1002/jrsm.1211 27322495

[B170] Steiger J. H. (1990). Structural model evaluation and modification: an interval estimation approach. Multivariate Behav. Res. 25, 173–180. 10.1207/s15327906mbr2502_4 26794479

[B171] ^*^ Stenling A. Ivarsson A. Hassmén P. Lindwall M. (2015). Using bifactor exploratory structural equation modeling to examine global and specific factors in measures of sports coaches' interpersonal styles. Front. Psychol. 6, 1303. 10.3389/fpsyg.2015.01303 26388808 PMC4555658

[B172] ^*^ Stenling A. Ivarsson A. Lindwall M. Gucciardi D. F. (2018). Exploring longitudinal measurement invariance and the continuum hypothesis in the Swedish version of the behavioral regulation in sport questionnaire (BRSQ): an exploratory structural equation modeling approach. Psychol. Sport Exerc. 36, 187–196. 10.1016/j.psychsport.2018.03.002

[B173] ^*^ Styck K. M. Rodriguez M. C. Yi E. H. (2021). Dimensionality of the state-trait inventory of cognitive and somatic anxiety. Assessment 29, 103–127. 10.1177/1073191120953628 32862664

[B174] ^*^ Summers J. J. Falco L. D. (2020). The development and validation of a new measure of adolescent purpose. J. Exp. Educ. 88, 47–71. 10.1080/00220973.2019.1575178

[B175] ^*^ Sutherland E. A. (2020). The measurement of job satisfaction among workplace leaders: Scale development and validation of the leader satisfaction assessment. (Doctoral thesis), Western University. Available online at: https://ir.lib.uwo.ca/etd/7006 (accessed June 29, 2021).

[B176] ^*^ Tomás J. M. Gutiérrez M. Alberola S. Georgieva S. (2022). Psychometric properties of two major approaches to measure school engagement in university students. Curr. Psychol. 41, 2654–2667. 10.1007/s12144-020-00769-2

[B177] ^*^ Tóth-Király I. Bothe B. Orosz G. (2017). Exploratory structural equation modeling analysis of the self–compassion scale. Mindfulness 8, 881–892. 10.1007/s12671-016-0662-1 PMC5681952 29163325

[B178] ^*^ Tóth-Király I. Böthe B. Orosz G. Rigo A. (2019). A new look on the representation and criterion validity of need fulfillment: application of the bifactor exploratory structural equation modeling framework. J. Happiness Stud. 20, 1609–1626. 10.1007/s10902-018-0015-y

[B179] ^*^ Tóth-Király I. Morin A. J. S. Böthe B. Orosz G. Rigó A. (2018). Investigating the multidimensionality of need fulfillment: a bifactor exploratory structural equation modeling representation. Struct. Equ. Model. 25, 267–286. 10.1080/10705511.2017.1374867

[B180] ^*^ Tóth-Király I. Morin A. J. S. Salmela-Aro K. (2021). Reciprocal associations between burnout and depression: An 8-year longitudinal study. Appl. Psychol. Int. Rev. 70, 1691–1727. 10.1111/apps.12295

[B181] ^*^ Trógolo M. A. Morera L. P. Castellano E. Spontón C. Medrano L. A. (2020). Work engagement and burnout: real, redundant, or both? A further examination using a bifactor modelling approach. Euro. J. Work Organ. Psychol. 29, 922–937. 10.1080/1359432X.2020.1801642

[B182] ^*^ Vajda D. Thege B. K. Rózsa S. (2019). Factor structure of the dyadic adjustment scale: a bifactor exploratory structural equation modeling approach. Euro. J. Psychol. Assess. 35, 326–334. 10.1027/1015-5759/a000405

[B183] ^*^ Van Zyl L. E. Olckers C. Roll L. C. (2020). The psychometric properties of the Grit–O scale within the twente region in Netherlands: an ICM–CFA vs. ESEM approach. Front. Psychol. 11, 796. 10.3389/fpsyg.2020.00796 32457679 PMC7223155

[B184] ^*^ Vaughan R. Madigan D. J. Carter G. L. Nicholls A. R. (2019). The dark triad in male and female athletes and non-athletes: group differences and psychometric properties of the short dark triad (SD3). Psychol. Sport Exerc. 43, 64–72. 10.1016/j.psychsport.2019.01.002

[B185] ^*^ Vaughan R. S. Edwards E. J. MacIntyre T. E. (2020). Mental health measurement in a post Covid-19 world: psychometric properties and invariance of the DASS−21 in athletes and non-athletes. Front. Psychol. 11, 590559. 10.3389/fpsyg.2020.590559 33192930 PMC7641904

[B186] ^*^ Volmer J. Koch I. K. Wolff C. (2019). Illuminating the 'dark core': mapping global versus specific sources of variance across multiple measures of the dark triad. Pers. Individ. Dif. 145, 97–102. 10.1016/j.paid.2019.03.024

[B187] ^*^ Wang T. Zhang T. Mu W. L. Li X. (2021). Psychometric evaluation of the encouragement character strength scale in a Chinese sample. Curr. Psychol. 40, 4741–4749. 10.1007/s12144-020-01161-w

[B188] ^*^ Yi Z. Y. Wang Y. Tan T. X. (2021). Evidence for a higher-order ESEM structure of ADHD in a sample of Chinese children. J. Psychopathol. Behav. Assess. 43, 376–387. 10.1007/s10862-020-09837-0

[B189] Zhu L. Wang J. Schroevers M. J. (2020). Looking beyond the value of individual facets of mindfulness: a person-centered examination of mindfulness. Mindfulness 11, 2349–2359. 10.1007/s12671-020-01452-0

